# Novel Insights into Understanding the Molecular Dialogues between Bipolaroxin and the Gα and Gβ Subunits of the Wheat Heterotrimeric G-Protein during Host–Pathogen Interaction

**DOI:** 10.3390/antiox11091754

**Published:** 2022-09-05

**Authors:** Deepti Malviya, Udai B. Singh, Budheswar Dehury, Prakash Singh, Manoj Kumar, Shailendra Singh, Anurag Chaurasia, Manoj Kumar Yadav, Raja Shankar, Manish Roy, Jai P. Rai, Arup K. Mukherjee, Ishwar Singh Solanki, Arun Kumar, Sunil Kumar, Harsh V. Singh

**Affiliations:** 1 Plant-Microbe Interaction and Rhizosphere Biology Lab, ICAR-National Bureau of Agriculturally Important Microorganisms, Kushmaur, Maunath Bhanjan 275103, India; 2 ICMR-Regional Medical Research Centre, Bhubaneswar 751023, India; 3 Department of Plant Breeding and Genetics, Veer Kunwar Singh College of Agriculture, Bihar Agricultural University, Dumraon 802136, India; 4 ICAR-Indian Institute of Vegetable Research, Varanasi 221305, India; 5 SRM University, Sonepat 131029, India; 6 ICAR-IIHR, Hessaraghatta Lake Post, Bengaluru 560089, India; 7 Department of Mycology and Plant Pathology, Institute of Agricultural Sciences, Banaras Hindu University, Varanasi 221005, India; 8 Division of Crop Protection, ICAR-National Rice Research Institute, Cuttack 753006, India; 9 Bihar Agricultural University, Bhagalpur 813210, India; 10 Centre for Agricultural Bioinformatics (CABin), ICAR-Indian Agricultural Statistics Research Institute, Library Avenue, PUSA, New Delhi 110012, India

**Keywords:** wheat, *Bipolaris sorokiniana*, Bipolaroxin, docking, molecular dynamics simulation, Agra Local, Chinese Spring, Chriya3, MAPK pathways

## Abstract

Spot blotch disease of wheat, caused by the fungus *Bipolaris sorokiniana* (Sacc.) Shoem., produces several toxins which interact with the plants and thereby increase the blightening of the wheat leaves, and Bipolaroxin is one of them. There is an urgent need to decipher the molecular interaction between wheat and the toxin Bipolaroxin for in-depth understanding of host–pathogen interactions. In the present study, we have developed the three-dimensional structure of G-protein alpha subunit from *Triticum aestivum*. Molecular docking studies were performed using the active site of the modeled G-protein alpha and cryo-EM structure of beta subunit from *T. aestivum* and ‘Bipolaroxin’. The study of protein–ligand interactions revealed that six H-bonds are mainly formed by Glu29, Ser30, Lys32, and Ala177 of G-alpha with Bipolaroxin. In the beta subunit, the residues of the core beta strand domain participate in the ligand interaction where Lys256, Phe306, and Leu352 formed seven H-bonds with the ligand Bipolaroxin. All-atoms molecular dynamics (MD) simulation studies were conducted for G-alpha and -beta subunit and Bipolaroxin complexes to explore the stability, conformational flexibility, and dynamic behavior of the complex system. *In planta* studies clearly indicated that application of Bipolaroxin significantly impacted the physio-biochemical pathways in wheat and led to the blightening of leaves in susceptible cultivars as compared to resistant ones. Further, it interacted with the Gα and Gβ subunits of G-protein, phenylpropanoid, and MAPK pathways, which is clearly supported by the qPCR results. This study gives deeper insights into understanding the molecular dialogues between Bipolaroxin and the Gα and Gβ subunits of the wheat heterotrimeric G-protein during host–pathogen interaction.

## 1. Introduction

Wheat, being the number one cereal in terms of production and consumption, needs special focus for enhancing or sustaining its productivity to feed the ever-growing human population. Spot blotch of wheat, caused by the hemi-biotroph fungal phytopathogen *Bipolaris sorokiniana* (Sacc.) Shoem., is now becoming a serious threat to wheat cultivation in warm, humid regions worldwide [1,2]. It ranks second after rusts in causing yield losses of wheat [3]. However, the incidence of different diseases with the evolution of new pathogenic races (such as ug99 for wheat rust) has made it difficult to manage the yield losses caused by them. In India, although incidences of spot blotch had been noticed as early as in 1924 [4], it has not been given due attention even to date. However, its importance in India was realized just after the Green Revolution, when almost all the wheat cultivars were showing susceptibility to this disease, which covered large areas. Thus, over the last four decades, spot blotch has been reported as a serious constraint in wheat production in the Eastern Gangetic Plains (EGP) of North India [3]. It is estimated that 25 million ha of the global wheat area is affected by the disease [5] and about 40% of this falls under the Indian subcontinent [3]. The crop losses due to spot blotch have been estimated to be in the range of 15–25% [6]. To control *B. sorokiniana,* chemical fungicides are being used at large scales, which is not an environmentally friendly practice. So, there is a need to develop specific and environment-friendly fungicides for which proper and in-depth understanding of host–pathogen interactions are needed. Further, *Bipolaris sorokiniana* produces a range of toxic compounds commonly known as phytotoxins, which include prehelminthosporol, helminthosporol, helminthosporic acid, and sorokinianin [7]. These compounds are known to inhibit seed germination and hypocotyl/radical growth [8]. Jahani et al. [7] identified a new compound, ‘Bipolaroxin’, which produces yellow and/or necrotic lesions when placed on the wheat leaves [7,9]. Cell membrane and membrane-bound receptors are the key targets of Bipolaroxin produced by *B. sorokiniana.* In general, Bipolaroxin at a higher concentration (>15 ng mL^−1^) impairs membrane integrity and biochemical pathways affecting the functionality of cellular organelles. It also generates a large amount of reactive oxygen species (ROS) in the cytosol, leading to quick cell death. Plants respond differently to different types of pathogens attacking wheat plants [10]. Now, it is becoming a challenging task to understand the plant immune system against *B. sorokiniana* and the toxin, Bipolaroxin, produced by them.

Recently, another important player behind the resistance to necrotrophic fungi was discovered, i.e., heterotrimeric G-protein [11,12,13]. Heterotrimeric G-proteins are universal signal transducers composed of α, β, and γ subunits coupled with a plethora of receptors commonly known as G-protein-coupled receptors (GPCRs) found in all eukaryotic organisms, including plants, fungi, and animals [14]. Moreover, the three different subunits of the heterotrimer, α, β, and γ, are organized in a highly conserved manner, which is bound to specific transmembrane G-protein-coupled receptors (GPCRs). Moreover, heterotrimeric G-protein signaling in plants differs from that in animals. Only 1 canonical Gα subunit gene has been identified in monocots and dicots, as compared to 17 in mammalian species [15]. The structure of the plant heterotrimeric G-protein does share important characteristics with its putative mammalian counterparts. The *Arabidopsis thaliana* gene *GPA1* encodes a Gα subunit (GPα1, 45 kDa), which is almost identical (36%) to Gαi1, a member of the mammalian Gi class of Gα subunits [16,17]. Although the existence of a heterotrimer in *Arabidopsis* has not yet been experimentally verified, the formation of heterotrimeric G-protein complexes was recently reported in the rice plasma membrane [18]. The plant heterotrimeric G-protein has been implicated in auxin, gibberellin, and abscisic acid signaling, light responses, cell division ion-channel regulation [19,20], and in the host–pathogen interaction [14]. Necrotrophic pathogens trigger Gα subunit and, in contrast, sometimes Gβγ dimmers [21]. Further, the Gβ was involved in defense against necrotrophic fungi such as *Botrytis cinerea, Alternaria brassisicola, Plectosphaerella cucumerina*, and vascular pathogen *Fusarium oxysporum* in Arabidopsis [10,12,21]. However, the Gα-subunit was involved in defense against the hemi-biotrophic pathogens such as rice blast pathogen *Magnaporthe grisea* in rice and *Arabidopsis* [22]. A number of reports suggest the direct roles of G-proteins in plant defense against a variety of pathogens [10,11,12,23]. However, the signaling role and host–pathogen interaction may be specific to each host–pathogen pair and therefore needs thorough investigation to develop a comprehensive understanding of the interaction taking place in the specific host–pathogen interaction. Trusov et al. [12] claimed that jasmonic acid (JA) was involved in heterotrimeric G-protein mediated resistance against necrotrophic pathogen in *Arabidopsis*. Later, the authors [22] demonstrated that G-protein mediated resistance to necrotrophic pathogens includes mechanisms independent of salicylic acid (SA), jasmonic acid/ethylene (JA/ET), and abscisic acid (ABA) signaling.

During the last few decades, many advances have occurred which enhance the understanding of plant–pathogen interactions. Further, the functional aspects of host–pathogen interaction have been reported using new generation omics research, i.e., advancement of genomics, transcriptomics, proteomics, and bioinformatics approaches. This will also facilitate the identification of molecular regulators as well as targets of signal transduction pathways involved in the resistant and susceptible cultivars. In our earlier publications, we have shown the involvement of jasmonate and bioagent *Trichoderma harzianum, Bacillus amyloliquefaciens,* and *Pseudomonas fluorescens* in the induction of systemic resistance (ISR and SAR) [1,2,24]. However, Bipolaroxin and its interaction with wheat (*Triticum aestivum* L.) plant have not been elucidated and needs in-depth investigation. Furthermore, the role and involvement of the Gα and Gβ subunits of the wheat heterotrimeric G-protein in wheat plant challenged with Bipolaroxin needs to be explored to find out the mechanism behind the host–pathogen interaction. The host–pathogen interaction mediated by cognate receptor and corresponding effector molecules can be explored through the molecular dynamics simulation (MD) studies [25,26,27,28]. Therefore, in the present study, we employed the molecular modeling technique coupled with MD simulations to infer the probable mode of binding of Bipolaroxin to the Gα and Gβ subunits and how Bipolaroxin modulates the physio-biochemical and molecular mechanisms in wheat. Statistical approaches involving principal component analysis (PCA), ensemble clustering, and molecular mechanics-based binding free energy calculations were also employed to elucidate the global motion and the energetic component that drives the interaction of the Gα and Gβ subunits with Bipolaroxin. Further, influence of Bipolaroxin on the disease development, physio-biochemical pathways, and regulation of downstream processes were deciphered in the present investigation. The results of this study offer initial understandings into the mode of binding and dynamics of Bipolaroxin with G-protein alpha subunits, which is expected to provide new avenues to the host–pathogen interaction.

## 2. Materials and Methods

### 2.1. Data Acquisition and Molecular Modeling of G-Protein Alpha Subunit

To date, the experimental structure of only one G-protein alpha subunit 3D structure (PDB ID: 2XTZ) from plants, i.e., the G Alpha Protein Atgpa1 from *Arabidopsis thaliana,* is available from Protein Data Bank (RCSB PDB). As such, to derive the 3D structure of the G-protein alpha subunit from *Triticum aestivum*, we first obtained the amino acid sequence from GenBank (GenBank Accession Number: BAC10502.1). We employed BLAST search against PDB to obtain suitable templates for theoretical modeling. The 3D structure of the subunit was determined using theoretical modeling based on two template structures, including 2XTZ (G alpha protein from *Arabidopsis thaliana*) and 3UMR (human G-alpha-i1). Based on the multiple-template approach, three-dimensional structures of the G-protein alpha subunit were modeled using Modeller9v19 [29,30]. Based on the Modeller scoring function, the best model was subjected to loop and side-chain refinement using Galaxy-refine [31] and What-If server (https://swift.cmbi.umcn.nl/whatif/ (accessed on 30 June 2022)). The optimized model was then subjected to stereo-chemical quality assessment using SAVES5.0, ProSA-Web [32], METAMQAPII, and ProQ [33]. The three-dimensional structure of the beta subunit was obtained from the cryo-EM structure of a small subunit RNA of *Triticum aestivum* (PDB ID: 4V7E_B).

### 2.2. Molecular Docking of ‘Bipolaroxin’

To obtain insights into the mode of binding of ‘Bipolaroxin’, we employed the molecular docking suite AutoDock4.2. Docking studies were performed on the active site of the modeled G-protein alpha and cryo-EM structure of the beta subunit from *Triticum aestivum*. Based on the structural superposition with the structural homologs, the ligand-binding residues were noted and compared with ligand-binding pocket identification tools HotSpot Wizard 3.0, CASTp [34], and GalaxySite (https://galaxy.seoklab.org/cgi-bin/submit.cgi?type=SITE (accessed on 30 June 2022)). The structure of Bipolaroxin was obtained from PubChem (CID: 146960) and optimized using Automated Topology Builder (ATB) and Repository Version 3.0. The following parameter was employed for docking in AutoDock; Lamarckian genetic algorithm with the number of runs: 300; population size: 150; the maximum number of evaluations: 25,000,000; the number of generations; 27,000; and the rate of cross over: 0.8. AutoDock scoring function (i.e., short-range van der Waals and electrostatic interactions, hydrogen bonding, and entropy losses) were included for binding energy estimation. The grid was set based on the consensus list of residues covering the ligand-binding pocket in the x, y, and z-axis directions with a grid spacing of 0.375 Å. The RMS cluster tolerance was set to 2.0 Å. Based on the RMSD-based clustering approach, the ligand binding poses were ranked using higher negative binding free energy with a greater number of H-bonds. AutoDock generated the lowest binding energy (most negative) docking conformation of Gα, and Gβ subunit-’Bipolaroxin’ complex was then subjected to protein–ligand interaction visualization using LigPlot^+^ and BIOVIA Discovery Studio Visulizer4.5 (BIOVIA DSV).

### 2.3. MD Simulations of G-Alpha and -Beta Subunit-‘Bipolaroxin’ Complex

Classical all-atoms MD simulations were conducted for G-alpha and -beta subunit-’Bipolaroxin’ complex using the Gromacs5.1 package using AMBER99SB force field. The topology parameter of the ligand was built using an ACPYPE server. Both the G-alpha and -beta subunit-‘Bipolaroxin’ complexes were immersed in a cubic box of TIP3P water models. A strength of 0.15M NaCl was added to neutralize both the systems. Energy minimization was performed using the steepest descent method of 10,000 steps followed by the conjugate gradient method for 10,000 steps to release conflicting contacts. Position-restrained dynamics simulations including NVT and NPT ensemble of the system were conducted at 300 K for 5 ns (nanosecond) at constant temperature and pressure followed by the production MD run for 50 ns. The long-range interactions were treated with the Particle Mesh Ewald (PME) method. The Lennard-Jones potentials were used for van der Waals interactions [35]. The distance cut-off of 10.0 Å was applied to calculate the nonbond interactions. The temperature and pressure of each system were kept constant at 300 K and 1 bar, respectively. The structural stability and dynamics parameter of the system was gauged from the resultant trajectory using built-in modules of GROMACS. The STRIDE program integrated with Visual Molecular Dynamics (VMDv1.9.2) was employed to analyze the time-dependent secondary structural analysis to perceive the secondary structure stability [36]. The dynamics stability parameters were computed using built-in modules of GROMACS and graphs were later plotted using XMGrace.

### 2.4. Clustering and Principal Component Analysis

To explore the conformational heterogeneity in the ensemble of both the complex systems produced by MD simulation, we employed RMSD-based clustering analysis with a cut-off of 0.25 nm using the GROMOS algorithm. Herein, the ‘*gmx cluster’* utility toolkit of GROMACS was used for structural cluster analysis by the method described by Daura and coworkers [37]. Principal component analysis (PCA) was carried out by generating a covariance-matrix of the atomic fluctuations. This covariance matrix was diagonalized to extract a set of eigenvectors and eigenvalues that reflect the combined motion of the molecule. Eigenvectors represent the direction of motion, whereas the corresponding eigenvalues represent the amplitudes in those directions. The GROMCAS in-built tool ‘*gmx covar*’ was used to extract the eigenvalues and eigenvectors of the MD trajectories extracted from each time frame of 50 ns MD trajectories, whereas the ‘*gmx anaeig’* toolkit was used to analyze and plot the eigenvectors [37].

### 2.5. MM/PBSA Binding Free Energy

Binding free energies calculations performed from the snapshots of MD trajectory using the molecular mechanics Poisson Boltzmann surface area (MM/PBSA) method implemented in the *g_mmpbsa* tool were well-suited to GROMACS trajectories. The binding free energy of the G-alpha and -beta subunit-’Bipolaroxin’ complexes were analyzed by taking 500 snapshots at a time interval of 30 to 50 ns (equilibrium phase) MD simulation. The protocol for binding free energy was adopted from our previous studies [36,38,39]. 

### 2.6. In Planta Study

#### Fungal Strains and Growth Conditions

While working on *Bipolaris sorokiniana*–wheat pathosystem [1,2,24], we explored different parts of Indo Gangetic Plains regions of India, collected diverse strains of *B. sorokiniana,* and preserved strains in the Plant–Microbe Interactions and Rhizosphere Biology Lab, ICAR-National Bureau of Agriculturally Important Microorganisms (ICAR-NBAIM), Kushmaur, India. For this study, a total of 18 potential isolates were collected from the Plant–Microbe Interactions and Rhizosphere Biology Lab, ICAR-NBAIM, Kushmaur. These strains were further maintained by subculturing on potato dextrose agar medium (HiMedia, India) at 27 ± 1 °C and preserved at 4 °C until further use. 

### 2.7. Plant Material and Creation of Artificial Epiphytotic Conditions 

Three contrasting lines, Agra Local (susceptible), Chinese Spring, and Chriya3 (resistant to spot blotch infection) were obtained from the Department of Plant Breeding and Genetics, Veer Kunwar Singh College of Agriculture (Bihar Agricultural University, Sabour), Dumraon, Bihar, India, and were evaluated in the glasshouse conditions in pots. The experiments were conducted from November to March (2019–20). Fertilizers were applied to supplement nitrogen, phosphorus, and potassium (NPK) in a proportion of 120:60:40. Soil moisture was maintained by sprinkling sterilized water on alternate days. The growing conditions were as follows: average mean temperature range of 22–26 °C, relative humidity of 75–78%, and a 14/11 h light/dark photoperiod. 

### 2.8. Extraction and Purification of Toxins

The monoconidial culture was raised and inoculum was prepared from 7-days-old culture plates using sterile distilled water [40]. Prior to extraction of toxins, pathogenicity of the isolates was confirmed by inoculating 15-day-old seedlings of wheat genotype Agra Local (susceptible check) as per the methods described by Jahani et al. [7]. For extraction of fungal toxins, each strain was inoculated into a flask containing 340 mL of Fries modified medium supplemented with yeast extract (0.1%), sucrose, and glucose, separately, and incubated at 25 ± 1 °C for three weeks. The quantitative analyses of toxins produced by different strains were carried out using HPLC (Shimadzu, Japan, C-18 column), taking purified compound as standard (‘Bipolaroxin’, Sigma-Aldrich, Burlington, MA, USA), as per the methods described by Jahani et al. [7]. 

### 2.9. Experimental Setup

The experiments consisted of seven treatments in five replications under glasshouse conditions. The treatments were: T_1_- Pathogen inoculation, T_2_- partially purified toxin, T_3_- Bipolaroxin 25 ng mL^−1^, T_4_- Bipolaroxin 50 ng mL^−1^, T_5_- Bipolaroxin 75 ng mL^−1^, T_6_- Bipolaroxin 100 ng mL^−1^, and T_7_- Untreated control. Glasshouse experiments were laid out in a randomized block design. After 45 days of sowing, the pathogen (*B. sorokiniana*) was inoculated by spraying the spore suspension containing 1.45 × 10^6^ CFU mL^−1^ during evening hours (1600) [24]. However, partially purified toxins and different doses of Bipolaroxin were infiltrated into the leaf lamina (100 µL/leaf) as per the methods described by Bashyal et al. [41]. 

### 2.10. Expression Study

The qPCR analyses were performed to see the changes in the expression profile of key genes of phenylpropanoid cascade and MAPK and WRKY transcription factors along with the heteromeric G protein, Gα, and Gβ in the wheat genotypes treated with *B. sorokiniana*, crude toxins, and different doses of Bipolaroxin at 7 days of inoculation under glasshouse conditions. Total RNA was extracted using RNA isolation kit (Agilent, Santa Clara, CA, USA) and cDNA was synthesized using cDNA synthesis kit (BioRAD, Hongkong, China) following the manufacturers’ instructions. The quality and quantity of cDNA were analyzed using a nanodrop spectrophotometer (Thermo Scientific, Waltham, MA, USA). Expression analyses were performed using gene-specific primers (Supplementary Tables S2–S4) using sybr green master mix (Agilent, India) on the BioRAD Real Time PCR System (Model: MJ MiniOpticon, BioRAD, India) according to Singh et al. [42]. Actin was taken as the internal control. The relative transcript levels were calculated using the 2^−ΔΔC^_T_ method [43].

### 2.11. Histopathological Study

For scanning electron microscopy, leaf samples were collected randomly from each treatment at 30 days under pathogen inoculation and infiltration of a partially purified toxin obtained from *B. sorokiniana* as well as chemically synthesized Bipolaroxin were brought to the laboratory and washed in running tap water. Thereafter, leaf samples were fixed in a mixture of formaldehyde (37%) and glutaraldehyde (2.5%) in a 1:1 ratio for 24 h at 4 °C. After 24 h of fixation, samples were kept in an osmium tetraoxide solution (HiMedia, India) for 12 h at room temperature (27 °C). The fixed samples were further dehydrated using a gradient of ethyl alcohol, i.e., 30, 50, 70, 90, and 100% (30 min each), and dried under vacuum. After dehydration, samples were coated with gold (20 nm) and visualized under Scanning Electron Microscope (Hitachi S-3400N, Hitachi, Chiyoda-ku, Tokyo, Japan) as described by Singh et al. [42].

### 2.12. Development of Disease Symptom and Lignin Production

The pathogen was inoculated as per the methods described by Singh et al. [1]. The inoculated plants were observed regularly for symptom development, and lesion length was recorded at 30 days of pathogen inoculation and infiltration of the partially purified toxin obtained from *B. sorokiniana* as well as chemically synthesized Bipolaroxin. Acid-soluble lignin content was estimated in the plant leaves as according to Fredrik and Elisabeth [44] with few modifications [1].

### 2.13. Statistical Analyses

The glasshouse experiments were laid out in a randomized block design. Data analyses were performed using statistical software SPSS version 16.0. The heat map was prepared using the online server ‘ClustVis: a web tool for visualizing clustering of multivariate data (BETA) (https://biit.cs.ut.ee/clustvis/ (accessed on 30 June 2022))’. However, Origin 9.0 and Microsoft Excel (Windows 10.0) were used to prepare graphs. 

## 3. Results

### 3.1. This Three-Dimensionl Structure of G-Alpha Subunit from Wheat

**Figure 1:**
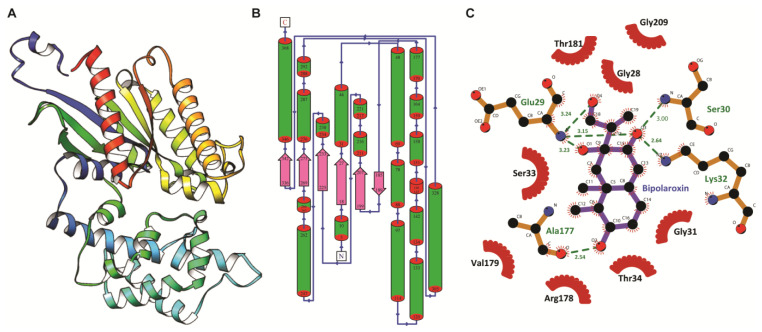
Three-dimensional model and 2D representation of the interaction between *Biopolaroxin* and G-Alpha subunit. (**A**) The solid ribbon representation of three-dimensional architecture of modeled G alpha subunit of wheat with domains (helical: lower half and Ras domain: upper half). (**B**) The topology of architecture of the modeled structure where the position of each helix and strand has been labeled from N-terminal end to c-terminal end. (**C**) Molecular representation of the top-ranked docked complex obtained from molecular docking of G-alpha subunit with Bipolaroxin rendered using LigPlot^+^ tool. The green dotted lines show the hydrogen bonds, residues with dark-red semicircles forming hydrophobic contacts, and residues labeled in green portray the H-bond forming amino acids.

For modeling the G-alpha and -beta subunit structure, the NCBI GenBank: BAC10502.1 and BAC10503.1, respectively, were chosen as the target sequence. In the case of the G-alpha subunit, based on the high percentage of sequence identity and coverage, putative templates, i.e., 2XTZ (identity of 74%) and 3UMR (identity of 37%) were used for the target–template alignment and subsequently used for modeling using the multi-template-based approach. Due to the lack of any structural information, the initial 20 residues (from the G-alpha subunit) were not considered for 3D modeling. The sequence alignment of the two templates with the target G-alpha subunit from wheat is shown in Figure S1. The model with the least-discrete optimized protein energy (DOPE) score along with the least Cα-root mean square deviation (RMSD) with the closest structural homolog 2XTZ was subjected to loop refinement and side chain optimization. Like the structural homolog, the modeled subunit was found to be comprised of two domains, i.e., the helical domain and the Ras domain (Figure 1A). Comparative analysis with the AtGPA1 (2XTZ) displayed minute differences in the variable loop region; however, the secondary structure elements were found to superpose well with a Cα RMSD of 0.63 Å. Among the secondary structure elements, the beta strand comprised 40 amino acids (10.8%), alpha helix comprised 165 amino acids (44.6%), 3_10_ helix formed 21 amino acids (5.7%), and turns/coils composed 144 residues (38.9%). Overall, the structure comprised 1 beta sheet, 2 beta-alpha-beta units, 1 beta hairpin, 1 psi loop, 6 strands, 20 helices, 29 helix–helix interactions, and 27 beta turns (Figure 1B).

### 3.2. Validation of G-Alpha Subunit Modeled Structure

**Figure 2:**
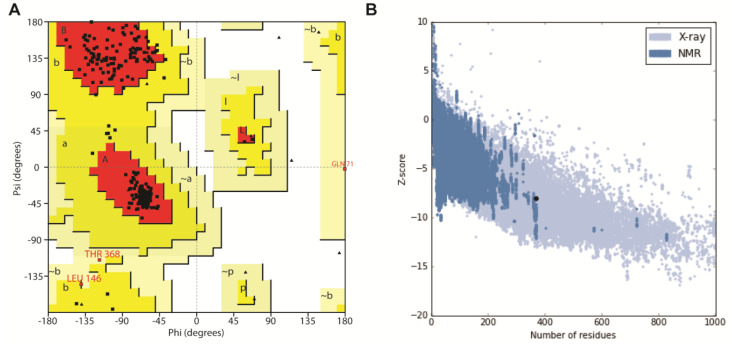
Ramachandran plot and ProSA z-score analysis of the modeled G-alpha subunit of *Triticum aestivum*. (**A**) The Ramachandran plot was generated using Procheck tool embedded in SAVES and the z-score plot was plotted using ProSA-Web (**B**).

The overall stereo-chemical qualities of the model were evaluated to warrant that it was suitable for carrying out further studies. Prior to starting the actual evaluation process, the active site residues of the G-alpha subunit were determined by structural superposition with the co-ordinates of the backbone Cα atoms of the modeled structure and X-ray crystal structure of G alpha protein AtGPA1 from Arabidopsis thaliana (2XTZ-A chain) using PyMOL. It was observed that the active site residues of the 2XTZ crystal structure (bound to the 5′-Guanosine-Diphosphate-Monothiophosphate) superposed well with our modeled structure, which indicates that active site residues were found to be more or less conserved in G-alpha subunits of plants. Stereo-chemical quality was explored using Ramachandran plot. Procheck was employed to validate the rationality of homology model. The Ramachandran plot analysis displayed the dihedral angles Φ against Ψ of amino acid residues in the predicted model, where 94% of the residues were found to be in the most favored region and none of the residues with bad geometry (outlier region of Φ and Ψ plot) depict good quality of the model (Figure 2A). ERRAT program was used to assess the overall quality factor of the modeled protein by determining the false statistics of bad, nonbonded interactions within the structure. The overall quality factor of the modeled structure was 86.76%, which indicates that the proposed model was of good quality. Additional analysis of the structure using the Profile-3D Verify score showed a comparatively good score of 82.22%, indicating the correctness of the homology model. Analysis of the predicted model using the ProQ server indicated the LG-score of “extremely good model” and Max-Sub of “very good model” quality measures. ProSA-Web analysis also displayed the energy profile and Z-score of the model (Figure 2B). The Z-score for the predicted model was found to be within the range of scores typically found for the native proteins of similar size, while the plot of energies of each residue revealed that the entire calculated value was negative. Overall, the quality evaluation using various model validation servers suggested that the generated model is reliable (Table 1) and thus can be used for docking studies with higher confidence.

### 3.3. Docking Analysis of ‘Bipolaroxin’

**Figure 3:**
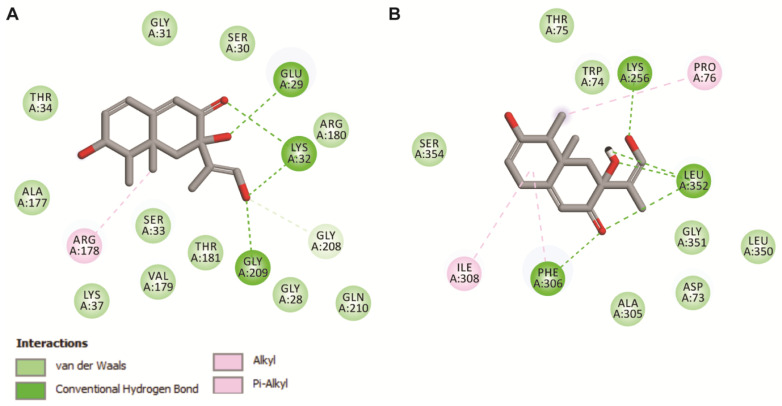
Intermolecular contact analyses of the top-ranked poses of Bipolaroxin with G-protein alpha (**A**) subunit and G-beta subunit of *Triticum aestivum* (**B**). The image was generated using BIOVIA DSV.

**Figure 4:**
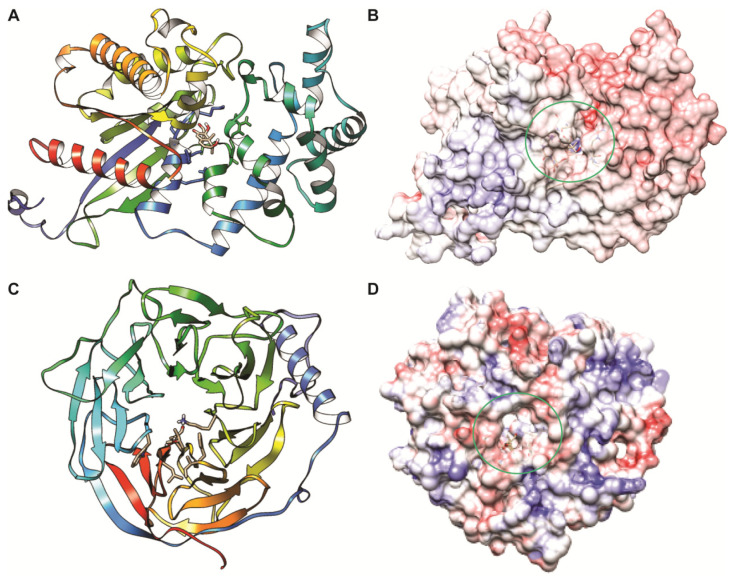
Docked conformational states and electrostatic surface representation of Bipolaroxin with G-protein alpha and G beta subunit. (**A**) Solid ribbon representation of the G-protein alpha subunit with Bipolaroxin (stick format) with the binding pocket residues. (**B**) Electrostatic surface potential map of G-protein alpha subunit with Bipolaroxin (ligand binding pocket has been marked in circle). (**C**) Solid ribbon representation of the G protein beta subunit with Bipolaroxin (stick format). (**D**) Electrostatic surface potential map of G protein beta subunit with Bipolaroxin (ligand binding pocket has been marked in circle). The electrostatic surface potential maps were generated using APBS and rendered using Chimera.

Molecular docking was performed to understand the mode of Bipolaroxin binding in modeled G-alpha and experimental beta subunit structures. The resulted docking conformations from the molecular docking experiment having lowest binding energy of −8.19 and −7.47 kcal/mol, respectively, were selected as the representative structure for protein–ligand interaction studies. Like that of a close structural homolog in the alpha subunit, the Bipolaroxin ligand prefers to bind in the cavity formed of helical and Ras domain of G-alpha subunit. Protein–ligand interaction study using LigPlot^+^ revealed six H-bonds mainly formed by Glu29, Ser30, Lys32, and Ala177 of G-alpha with Bipolaroxin (Figure 1C). In the beta subunit, the residues of the core beta strand domain participate in ligand interaction, where Lys256, Phe306, and Leu352 formed seven H-bonds with the ligand ‘Bipolaroxin’. In addition to H-bonds, hydrophobic contacts were also noticed to be formed of Gly28, Gly31, Ser33, Thr34, Arg178, Val179, Thr181, and Gly209 of G-alpha and Trp74, Ala305, Phe306, Ile308, Leu350, Gly351, and Ser354 of G-beta with Bipolaroxin (as displayed in Figure 1C, Figure 3 and Figure 4, and Table 2). We also computed the electrostatic surface potential of each complex using APBS plug-in in Chimera (as shown in Figure 4) which depicted that the ligand binding pocket is mostly dominated by a patch of negatively charged residues. 

### 3.4. MD Simulation

**Figure 5:**
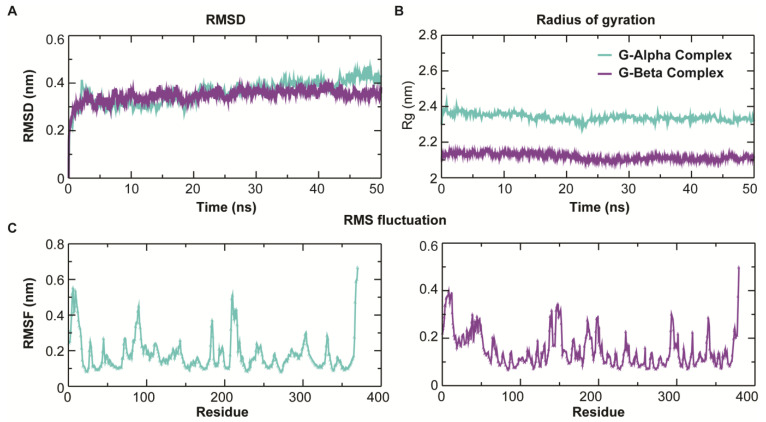
Intrinsic dynamics stability parameters of the G-alpha and G-beta subunit Bipolaroxin complexes during 50 ns MD simulation. (**A**) Root mean square deviation (RMSD) of backbone atoms of the modeled G-alpha subunit and experimental beta subunit in complexes with Bipolaroxin during 50 ns MD. (**B**) The compactness of the trajectory by calculating the radius of gyration (R_g_) of the proteins during 50 ns MD in aqueous solution. (**C**) The root mean square fluctuation (RMSF) for Cα atoms of the G-alpha (left) and G-beta (right) complex systems.

To measure the dynamic stability and conformational flexibility of the protein–ligand complexes, an all-atoms MD simulation study was conducted using GROMACS. To determine the stability and mechanistic aspects of the Bipolaroxin with the Gα and Gβ subunits of wheat interactions, a wide range of structural stability parameters were computed from the resultant MD trajectories using modules available in GROMACS. RMSD measures the difference between the backbones of a protein from its initial structural conformation to its final position. The stability of the protein relative to its conformation can be obtained by plotting the deviations produced during MD simulation. As a principle, the lesser the deviations, the more stable the protein–ligand complex system is. The RMSD of all starting protein–ligand configurations increased during the equilibration phase and converged after ~30 ns. The RMSD plot of both the systems displayed that the systems equilibrated after 30 ns and converged afterward with a stable RMSD of ~0.33 nm until 50 ns (as shown in Figure 5A). Both the systems displayed more or less the same trend in RMSD with minute exceptions during the last 5 ns for the Gβ system. The compactness of both the systems during the simulation was gauged by computing the radius of gyration (R_g_). Unlike RMSD, the trend in R_g_ profile also displayed a differential trend where the Gα subunit complex displayed a slightly higher R_g_ of ~2.35 nm (Figure 5B), while a more compact gyradius was observed in the case of the Gβ subunit complex (with an average R_g_ of ~2.1 nm). During the equilibration phase, the Rg was slightly on the higher side (during the initial 30 ns); however, in the later phase, a constant trend in Rg was observed indicating the stability and compactness of both the systems. The Cα-RMSF (root mean square fluctuations) per residue were calculated to determine protein regions exhibiting higher flexibility. Both the systems displayed differential flexibility, which is obvious due to differences in structural architecture. In the case of the Gα flexible residue, it was positioned at 20–40, 110–130, and 205–235 with higher peaks (Figure 5C). Visual inspection of these regions revealed that most of the residues were in the loop regions and the ligand binding pocket had a great degree of fluctuations, indicating that the protein shows acceptability to other molecules and adopts conformational changes based on interactions. Further, to confirm the stability of the protein–ligand systems, the DSSP algorithm was used to evaluate the changes in secondary structure during 50 ns MD simulations. In this study, there were no significant changes in structural elements observed during the entire simulation time except for the unstable 3_10_ helices in the Gα system (as shown in Figure S2A), which affirms the stability of the complex system. However, in the case of the Gβ system, we observed a significant change in the turns connecting the core of the beta-strands (Figure S2B).

### 3.5. Essential Dynamics

**Figure 6:**
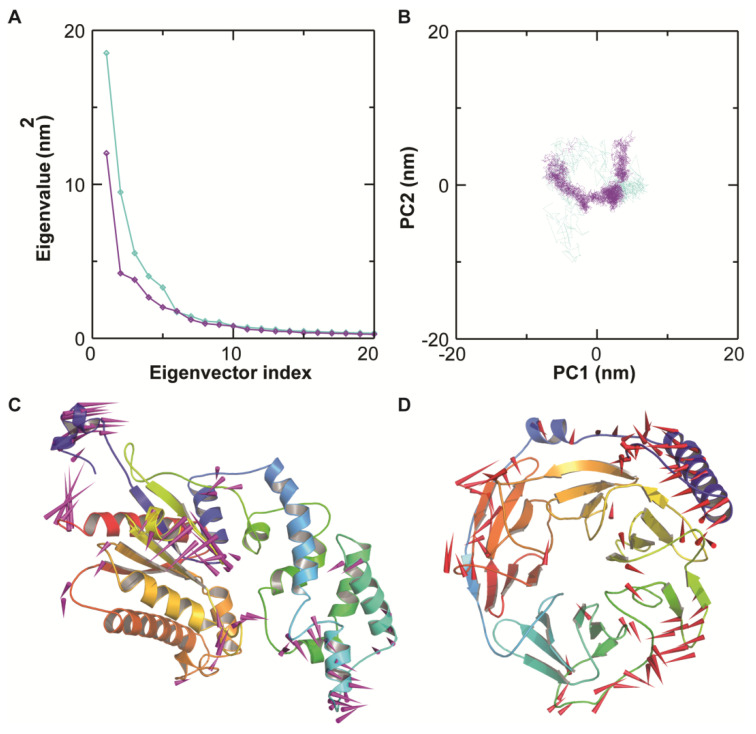
PCA of the protein–ligand (Gα and Gβ) systems using the resultant MD trajectories. (**A**) Eigenvalues for the complex as a function of the first 20 eigenvectors. (**B**) The cloud epitomizes the 50 ns trajectories projected onto the first two PCs where the x-axis and y-axis show the projection of the structures of the main-chain atoms in the MD trajectories onto the phase space defined by first two sets PCs (PC1 vs. PC2). (**C**) Porcupine plot depicting the movement of main-chain atoms of the first PC (PC1) of the G-α- Bipolaroxin complex. (**D**) Porcupine plot depicting the movement of main-chain atoms of the first PC of the G-β- Bipolaroxin complex. The direction of arrows indicates the motion and thickness of the arrow indicates the strength of motion. The image was generated using modevector.py script in PyMOL.

PCA analysis was performed on the MD trajectories to understand the global motion and conformational changes in the protein–ligand complex. By the diagonalization of the covariance matrix of the Cα atomic fluctuations, a set of eigenvalues were obtained and plotted with decreasing order versus the corresponding eigenvector indexes (as shown in Figure 6A). The first few eigenvalues corresponding to concerted motions quickly decrease in amplitude to reach a number of constrained and more-localized fluctuations. Specifically, the first two eigenvectors or principal components (PCs) obtained from PCA capture more than 81.73% (for Gα) and 87.9% (Gβ) of the total motion, indicating that these vectors define the essential subspace of the system. The scatter plot generated for the protein–ligand complex indicated the overall motion of the system was varied in nature (Figure 6B). The trace values of covariance matrices of the Gα complex were found to be 61.10 nm^2^, while for the beta subunit it was computed to be 43.46 nm^2^. This signifies the fact that the alpha subunit occupies more global conformational space than that of the beta subunit, with the least motion (Figure 6B). To understand the kind of motion governed by the PCs, we also generated the porcupine plots of the first PC (as shown in Figure 6C,D). In the G-alpha system, we observed a large outward movement in the beta-dominated region of the RAS domain along with small movements in the loop regions. Similarly, the loops connecting adjacent beta-strands in the core and surface region of the β subunit displayed a high degree of flexibility, which perfectly corroborates the results of RMSF analysis. The cross-correlation matrix of the Cα displacement indicated that both correlated and anticorrelated motions exist in both the systems (Figure S3). Highly positive regions (red) indicate a strong correlation in the movement of residues, whereas negative regions (blue) are associated with the strong anticorrelated motion of the residues. The colour depth of the diagonal represents the movement degree of atoms. The magnitude of the correlation was quantified by calculating the cross-correlation coefficient between the atomic displacements. The higher the absolute cross-correlation value, the better the two atoms are correlated (or anticorrelated).

### 3.6. Clustering Analysis

**Figure 7:**
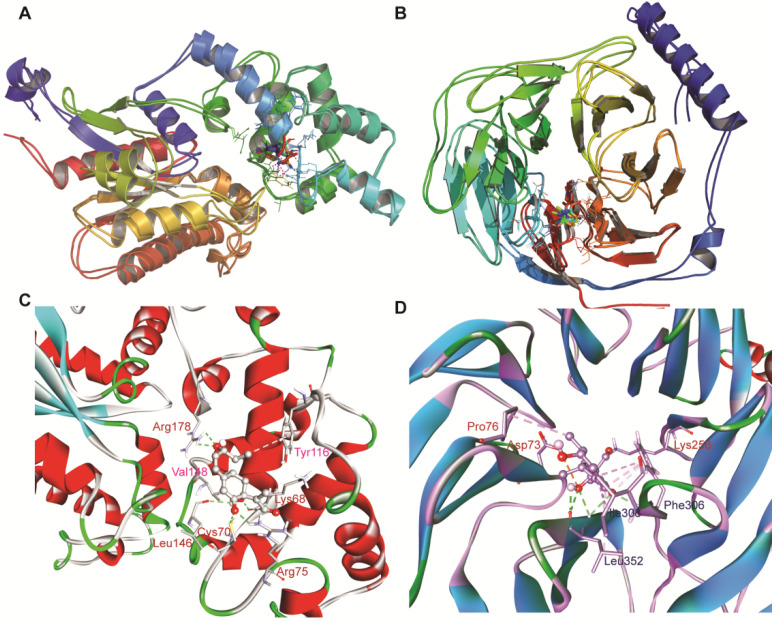
Structural superimposed view of the top two structural ensemble (top-ranked two clusters obtained from clustering). (**A**) Superimposed architecture of top-ranked two clusters from G-alpha subunit complex. (**B**) Superimposed architecture of top-ranked two clusters from G-beta subunit complex. Both the images were rendered using PyMOL. (**C**) Intermolecular contact of top-ranked cluster of Bipolaroxin with Gα subunit where the H-bond forming residues are marked in red while other nonbonded contacts are in pink. (**D**) Protein–ligand interaction analysis of the top-ranked cluster of Bipolaroxin with Gβ subunit where the H-bond forming residues are marked in blue while other nonbonded contacts are in red. The image was generated using BIOVIA DSV.

Cluster analysis was performed using the GROMOS method with an RMSD cut-off of 0.25 nm to understand the structural heterogenity in the ensembles during MD. The RMSD was calculated for the heavy atoms of the ligand after alignment of the Cα of the protein. All structures, i.e., the conformations at each time step, under 0.25 nm were clustered together, and the structure within the cluster with the most neighbors (i.e., the most similar structures), was set as the preliminary cluster centre. Then, the clusters, and their structures, were removed from the structure pool and a new cluster was determined and analyzed. The structure corresponding to the center of a cluster was determined and used as the predicted binding pose for the ligand. The cluster sizes were then ranked against each other and the cluster containing the most structures became the top-pose cluster. Based on the results of clustering, the top-two ranked clusters with a maximum of structures were considered for structural superposition (as shown in Figure 7A,B). The reasoning was that the largest cluster’s corresponding structure is probably one of the more energy-favorable poses, as it is the most-sampled pose during the MD simulation, and thus, probably the most accurate prediction with the lowest free energy. The top-pose clusters considered a significant cluster in both the systems were found to be comprised of 423 structures (84.6%) for Gα and it comprised of 467 (93.4%) for Gβ. As shown by Figure 7, it can be observed that in most of the systems, the ligand-binding pocket residues superpose well with a minor change in the orientation of the ligand. The top-ranked cluster for each system was investigated in detail in order to understand the molecular interaction with ligand Bipolaroxin (Figure 7C,D).

### 3.7. MM/PBSA Binding Free Energy

A total of 500 structures extracted (at equal intervals of time) from the equilibrated portion (30–50 ns) of the MD trajectory were used to compute MM/PBSA binding free energy of the protein–ligand complex systems. In order to obtain a deeper understanding of the contribution of different energy terms to the total binding free energy, individual energy components were inferred (as summarized in Table 3). According to a comparative analysis of the individual components contributing to the binding free energy, it can be concluded that van der Waals and electrostatic energy dominate the change in the binding strength. As compared to the van der Waals energy, there was a drop in the contributions from electrostatic terms, a fact that can be explained by a few hydrogen bonds formed between the protein and ligand during MD simulation. The total free energy calculations were also in agreement with the speculation above, indicating that nonpolar interactions, especially the van der Waals interactions, were the major contributors to the bonding of the G alpha and beta subunit with Bipolaroxin.

### 3.8. Probable Mode of Binding of ‘Bipolaroxin’

**Figure 8:**
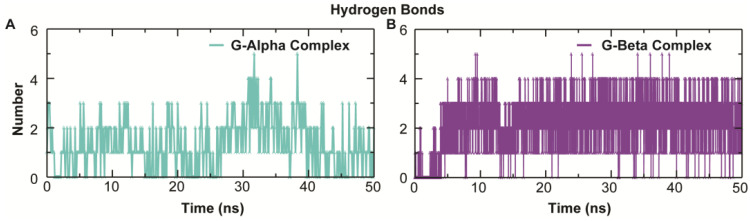
Dynamics of H-bonds between G-α (**A**) and β (**B**) subunit unit with ligand (Biploaroxin) complex systems with respect to time over the time scale of 50 ns.

Using the gmx hbond toolkit in GROMACS, the number of intermolecular hydrogen bonds (H-Bonds) between the protein and ligand (Bipolaroxin) during MD simulation was determined to understand the stability of both the systems. The H-bonding pattern was found to be unstable for the Gα system because the number of H-bonds constantly fluctuated throughout the MD simulation, while for Gβ, it was stable after the system attained equilibrium (Figure 8). Close inspection of the docked and cluster representative structure displayed that the H-bonds at the docking level were not maintained during the MD simulations mostly in the G-alpha system. However, a number of crucial H-bonds were observed to be broken in the course of the MD simulation that were later well-compensated through new H-bonds and hydrophobic contacts (as shown in Table S2). The observed differences in H-bond formation may be due to lateral movement of the ligand within the binding pocket. As evident from the protein–ligand interaction of the representing structure for the G-alpha system obtained from the top-ranked cluster, Arg178, leu146, Cys70, Arg75, and Lys68 formed strong H-bonds with the ligand, whereas Lys68, Val148, and Tyr116 formed hydrophobic contacts (alkyl and pi-alkyl types of interactions) with the ligand Bipolaroxin (Figure 7C,D and Table S2). In the case of the Gβ subunit system, Lys256, Phe306, and Leu352 formed strong H-bonds, while Phe306, Pro76, Ile308, and Leu352 alkyl and pi-alkyl hydrophobic made contact with the ligand (Table S2). In addition, Asp73 formed electrostatic contact with the oxygen (O1) atom of ‘Bipolaroxin’. Considering the fact that electrostatic and van der Waals interactions dominate the change in binding free energy, we also inferred the per-residue decomposition (as summarized in Figure S4). The per-residue decomposition of the overall binding energy analysis also suggested that most of the ligand-binding residues contributed to the overall negative free energy of each system.

### 3.9. In Planta Assay

In silico analyses clearly indicated that Bipolaroxin interects with the heteromeric G protein (Gα and Gβ) and modulates the structure which might impact the sensing of the toxin. Due to this, entire downstream signaling processes may become altered and break down the resistance mechanisms. Results of the in silico study prompt us to investigate the interaction of Bipolaroxin with heteromeric G protein (Gα and Gβ) and downstream processes in the susceptible (Agra Local) and resistant lines/cultivars (Chinese Spring and Chirya3). Bipolaroxin significantly affects the cellular processes and physiological and metabolic functions, and modulated several pathways/cascades in wheat, leading to program cell death and disintegration of the plant tissues. In the present investigation, the interaction of Bipolaroxin with heteromeric G protein (Gα and Gβ) modulates downstream signaling affecting the expression profile of key genes involved in the MAPK pathways, phenylpropanoid, and several other transporters leading to induced resistance/susceptibility in the resistance and sustainable genotypes.

### 3.10. Selection of Pathogenic Strain

**Figure 9:**
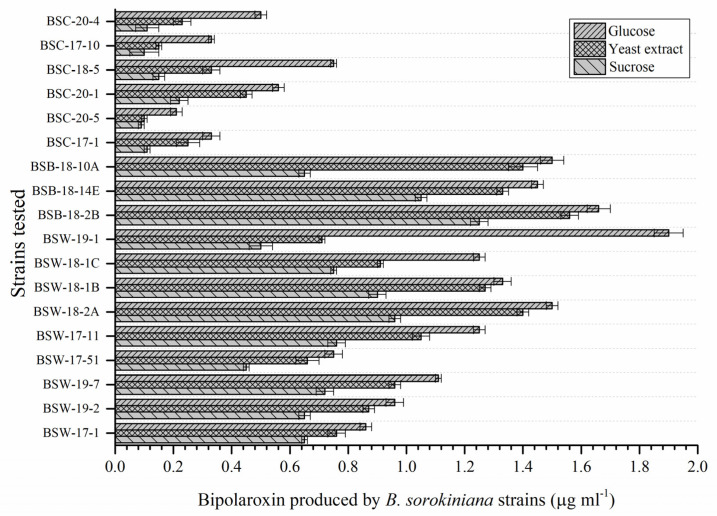
Production of Bipolaroxin by different strains of *B. sorokiniana* on the minimal media supplemented with sucrose, yeast extract, and glucose under in vitro laboratory conditions. Data are mean (*n* = 5) and vertical lines represent the standard deviation (Mean ± SD).

In the present investigation, 18 pathogenic strains were screened for production of Bipolaroxin on the minimal media supplemented with sucrose, yeast extract, and glucose under in vitro laboratory conditions. These strains were able to produce Bipolaroxin in a range of 0.09 to 1.66 µg mL^−1^ (Figure 9). A significantly higher amount of Bipolaroxin was produced by *B. sorokiniana* BSB-18-2B (1.66 µg mL^−1^) and followed by *B. sorokiniana* BSW-18-2a (1.50 µg mL^−1^) and *B. sorokiniana* BSB-18-10a (1.50 µg mL^−1^) in the liquid broth supplemented with glucose. However, the least amount of Bipolaroxin was produced by *B. sorokiniana* BSC-20-5 (0.09 µg mL^−1^) in the liquid broth supplemented with sucrose. In general, it was also observed that supplementation of glucose enhanced the production of Bipolaroxin significantly (Figure 9). Hence, glucose was taken as a potential supplement and *B. sorokiniana* strain BSB-18-2B was selected for further in planta investigation. 

### 3.11. Expression Profile of Genes Conferring Plant Defense

#### 3.11.1. Effects on Expression Profile of Gα- and Gβ Subunits of Heteromeric G Protein

**Figure 10:**
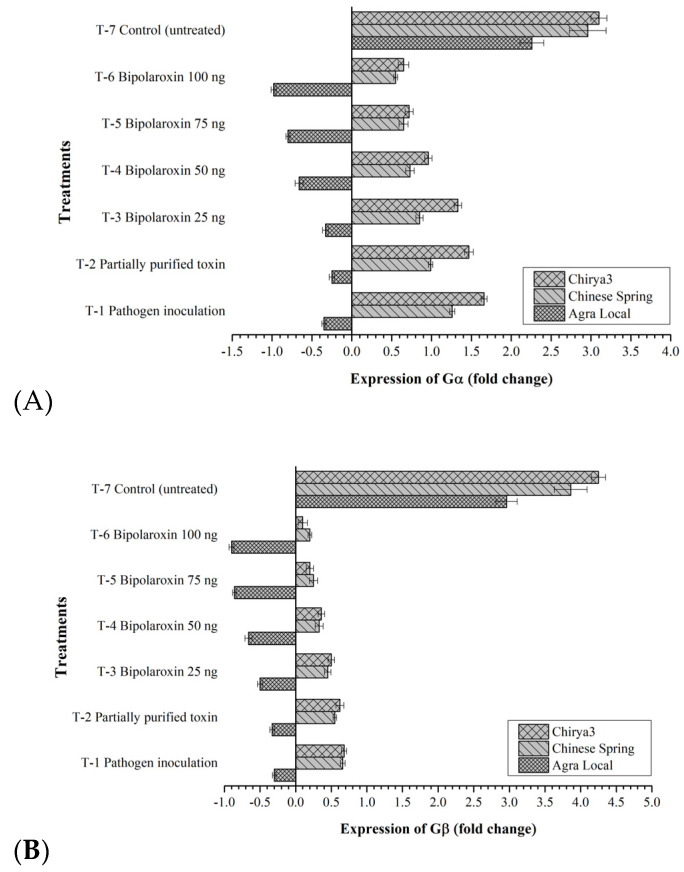
Expression profile of Gα (A) and Gβ (B) in susceptible line, Agra Local and resistant lines, Chinese Spring, and Chirya3 under pathogen inoculation and infiltration of partially purified toxin obtained from *B. sorokiniana* as well as chemically synthesized Bipolaroxin at 7 days of inoculation/treatments under glasshouse conditions. Data are mean (*n* = 3) and vertical lines represent the Standard Deviation (Mean ± SD).

In the past, several reports indicated the differential responses of the heterotrimeric G-protein subunits against a variety of phytopathogens including bacterial pathogens, vascular pathogens, biotrophic, hemibiotrophic, and necrotrophic fungi, including oomycetes. In the present study, we studied the effects of pathogen inoculation and infiltration of partially purified toxin obtained from *B. sorokiniana* as well as chemically synthesized Bipolaroxin on the expression of Gα and Gβ subunits of the wheat heterotrimeric G-protein in resistant and susceptible breeding lines. Quantitative real-time qPCR analyses clearly indicated that pathogen inoculation and infiltration of partially purified toxin and different concentrations of Bipolaroxin significantly impact the expression and transcript accumulation of Gα and Gβ subunits of the wheat heterotrimeric G-protein in susceptible and resistant lines. The results clearly authenticated a positive role of the genes/QTLs responsible for resistance in wheat germplasm. The Gα transcript accumulation was significantly lower in the susceptible line Agra Local, and maximum reduction was recorded in the plants infiltrated with Bipolaroxin 100 ng (−0.98 folds) followed by 75 ng (−0.80 folds) and 50 ng (−0.66 folds) as compared to the untreated control (2.26 folds). In contrast, the impact of pathogen inoculation and infiltration of partially purified toxin as well as chemically synthesized Bipolaroxin on the expression profile of Gα in resistant lines, Chinese Spring, and Chirya3 was significantly less as compared to the susceptible line Agra Local. The lowest transcript level of Gα was recorded in the plants infiltrated with Bipolaroxin 100 ng (Chinese Spring: 0.55 folds and Chirya3: 0.66 folds), followed by 75 ng (Chinese Spring: 0.65 folds and Chirya3: 0.72 folds) and 50 ng (Chinese Spring: 0.73 folds and Chirya3: 0.96 folds), as compared to the untreated control (Chinese Spring: 2.96 folds and Chirya3: 3.10 folds) and other treatments (Figure 10A). A similar trend was observed in the case of Gβ transcript accumulation in the plants under different treatments (Figure 10B). The results confirmed that Bipolaroxin played key roles in the signaling, downstream regulation, and pathogenicity of *B. Sorokiniana* on wheat.

#### 3.11.2. Effects on Expression Profile of Key Genes of Phenylpropanoid Pathway 

**Figure 11:**
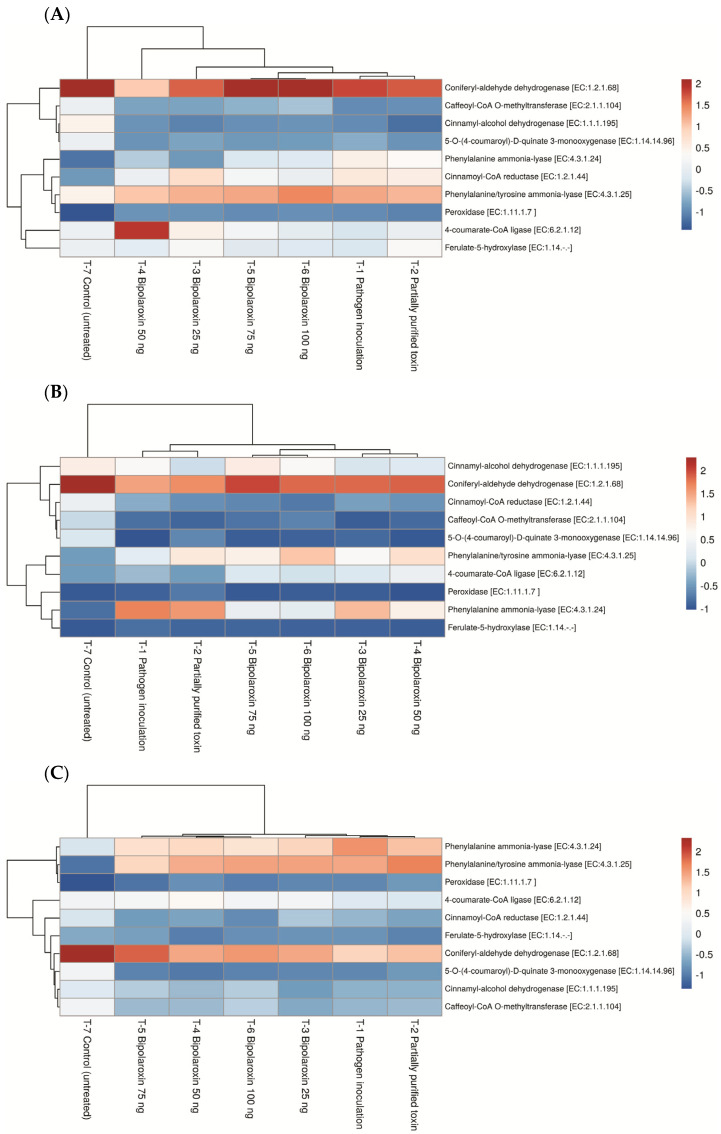
Expression profile of key genes of phenylpropanoid pathways in susceptible line, Agra Local (**A**) and resistant lines, Chinese Spring (**B**), and Chirya3 (**C**) under pathogen inoculation and infiltration of partially purified toxin obtained from *B. sorokiniana* as well as chemically synthesized Bipolaroxin at 7 days of inoculation/treatments under glasshouse conditions.

Phenylpropanoid is the key pathway playing an important role in plant responses to biotic and abiotic stresses. The role of phenylpropanoid cascades has traditionally been linked to the infection caused by either biotrophic, hemibiotrophic, and necrotrophic pathogens. In the present investigation, 10 key genes of phenylpropanoid pathway, i.e., phenylalanine ammonia-lyase [EC:4.3.1.24], phenylalanine/tyrosine ammonia-lyase [EC:4.3.1.25], 4-coumarate-CoA ligase [EC:6.2.1.12], cinnamoyl-CoA reductase [EC:1.2.1.44], cinnamyl-alcohol dehydrogenase [EC:1.1.1.195], peroxidase [EC:1.11.1.7 ], ferulate-5-hydroxylase [EC:1.14.-.-], caffeoyl-CoA O-methyltransferase [EC:2.1.1.104], coniferyl-aldehyde dehydrogenase [EC:1.2.1.68], and 5-O-(4-coumaroyl)-D-quinate 3-monooxygenase [EC:1.14.14.96] were taken into expression study. In general, all these genes were upregulated in the plants under treatments. In the case of susceptible line Agra Local, maximum expression was recorded for coniferyl-aldehyde dehydrogenase followed by phenylalanine/tyrosine ammonia-lyase and 4-coumarate-CoA ligase. However, the lowest expression was recorded in peroxidase as compared to other genes of tested phenylpropanoid pathways (Figure 11A). Among different treatments, significantly higher expression of all the genes tested was recorded in the plants treated with Bipolaroxin 100 ng, followed by 75 ng and 50 ng, as compared to other treatments. However, the lowest expression was recorded in untreated control plants (Figure 11A).

In contrast, a significantly higher expression of all these genes was recorded in the resistant cultivars, Chinese Spring, and Chirya3, as compared to the susceptible one, Agra Local, across the treatments. In the case of resistant line, Chinese Spring, maximum expression was recorded for coniferyl-aldehyde dehydrogenase, followed by phenylalanine/tyrosine ammonia-lyase, Cinnamyl-alcohol dehydrogenase, and phenylalanine ammonia-lyase. However, the lowest expression was recorded in peroxidase as compared to other genes of phenylpropanoid pathways tested (Figure 11B). A more or less similar pattern was recorded in the case of resistant line Chirya3, where maximum expression was recorded for coniferyl-aldehyde dehydrogenase, followed by phenylalanine/tyrosine ammonia-lyase, phenylalanine ammonia-lyase, and 4-coumarate-CoA ligase. However, the lowest expression was recorded in peroxidase and 5-O-(4-coumaroyl)-D-quinate 3-monooxygenase, as compared to other genes of phenylpropanoid pathways tested (Figure 11C). Among different treatments, the trends were similar to Agra Local (Figure 11A).

#### 3.11.3. Effects on Expression Profile of Key Genes of MKK/MAPK Pathways 

**Figure 12:**
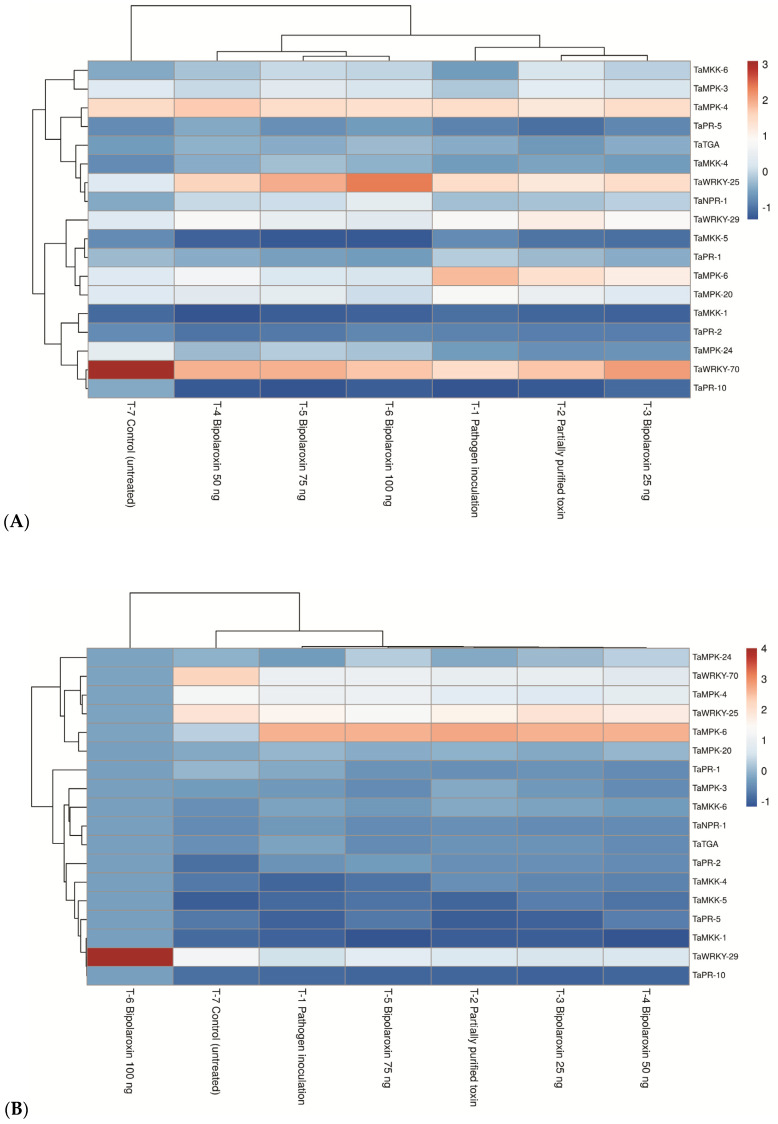

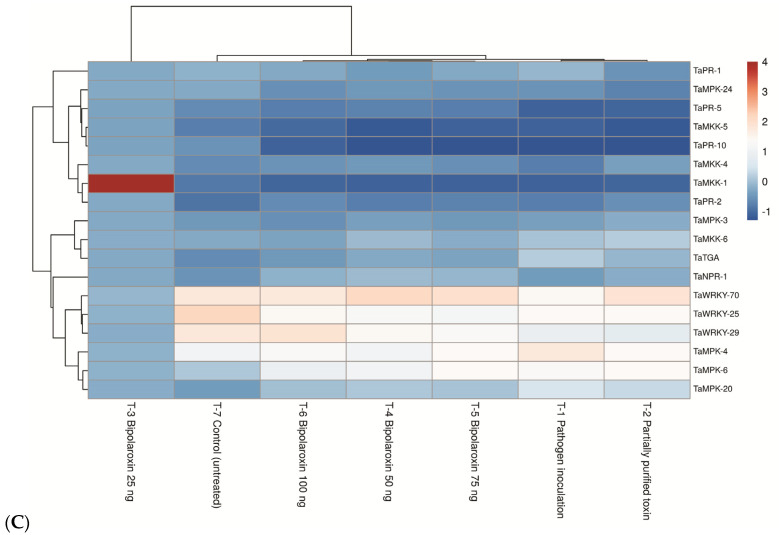
Expression profile of key genes of MKK/MAPK pathways in susceptible line, Agra Local (**A**) and resistant lines, Chinese Spring (**B**), and Chirya3 (**C**) under pathogen inoculation and infiltration of partially purified toxin obtained from B. sorokiniana as well as chemically synthesized Bipolaroxin at 7 days of inoculation/treatments under glasshouse conditions.

Further, the MKK/MAPK pathway is an important pathway playing a crucial role in signaling, activation of downstream cellular processes, and finally, elicitation of systemic resistance under biotic and abiotic stresses in the plant system. The role of the MKK/MAPK pathway, in general, started just after sensing the stress, effector molecules produced by either biotrophic, hemibiotrophic, and necrotrophic pathogens, or the presence of any foreign entity in/on the plant system. To determine whether the MKK/MAPK pathway is involved in Bipolaroxin–wheat interaction, the expression of several key genes of the MKK/MAPK pathway was investigated using real-time quantitative PCR (qRT-PCR) in susceptible and resistant lines of wheat. In the present investigation, 18 key genes of the MKK/MAPK pathway, viz., MKK1, MKK4, MKK5, MKK6, MPK3, MPK4, MPK6, MPK20, MPK24, WRKY23, WRKY29, WRKY70, NPR1, TGA, PR1, PR2, PR5, and PR10, were selected for expression study. The qRT-PCR analysis clearly indicated that MKK1 and MKK5 were significantly repressed in the leaves of susceptible line, Agra Local, infiltrated with Bipolaroxin 100 ng, followed by 75 ng and 50 ng, as compared to other treatments. However, all other genes were upregulated in the plants under treatments. TaWRKYs are known to be key regulators in the SA signaling pathway and to be involved in the SA analog-induced resistance in wheat against *B. sorokiniana* infection. In the case of susceptible line, Agra Local, maximum expression of WRKY23 was recorded in the plants treated with Bipolaroxin 100 ng (10.50-fold), followed by 75 ng (8.05-fold) and 50 ng (6.10-fold), as compared to other treatments. However, the lowest expression was recorded in the untreated control (10.50-fold) plants (Figure 12A). In general, the fold change in the transcripts was significantly lower in the susceptible line, Agra Local, as compared to the resistant lines, Chinese Spring and Chirya3 (Figure 12B,C). 

TaPR is generally seen as a systemic acquired resistance (SAR) marker gene and it has shown to be a pathogen/elicitor-inducible in many crops, including wheat. The transcription of NPR1, TGA, PR1, PR2, PR5, and PR10 significantly increased after pathogen inoculation or toxin infiltration even in the susceptible lines of wheat (Figure 12A). In contrast, the expression of MKKs and MPKs significantly increased (1.25- to 16.76-fold) in the leaves of resistant lines, Chinese Spring and Chirya3, infiltrated with Bipolaroxin 100 ng, followed by 75 ng and 50 ng, as compared to other treatments. The lowest expression of these genes was recorded in the untreated control plants (Figure 12B,C). Among resistant lines, in general, a significantly higher expression of all the genes was recorded in Chirya3. Similarly, transcript accumulation of TaWRKY23, TaWRKY29, and TaWRKY70 was significantly higher in the leaves of Chirya3 infiltrated with Bipolaroxin 100 ng (16.76-fold), followed by 75 ng (11.75-fold) and 50 ng (10.00-fold), as compared to other treatments (Figure 12C).

Similar to MKKs and MAPKs, significantly higher expressions of NPR1, TGA, PR1, PR2, PR5, and PR10 genes were recorded in the resistant cultivars Chinese Spring and Chirya3 as compared to the susceptible one, Agra Local, across the treatments. In the case of resistant line Chinese Spring, maximum expression was recorded for NPR1, followed by PR2, PR1, and TGA in the leaves of plants infiltrated with Bipolaroxin 100 ng, followed by 75 ng and 50 ng, as compared to other treatments (Figure 12B). However, the lowest expression was recorded in untreated control plants (Figure 12B). A more or less similar pattern was recorded in the case of resistant line Chirya3, where maximum expression was recorded for NPR1, followed by PR1, TGA, and PR2 (Figure 12C).

#### 3.11.4. Effects on Expression Profile of Antioxidant Genes 

**Figure 13:**
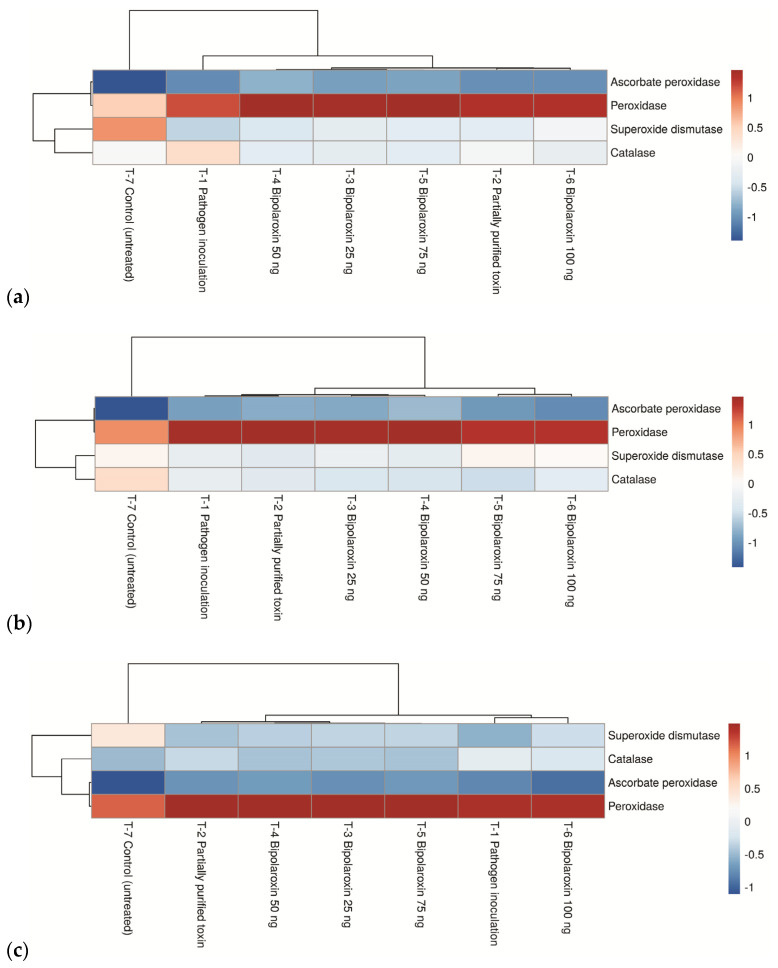
Expression profile of antioxidant genes in susceptible line, Agra Local (**a**) and resistant lines, Chinese Spring (**b**), and Chirya3 (**c**) under pathogen inoculation and infiltration of partially purified toxin obtained from *B. sorokiniana* as well as chemically synthesized Bipolaroxin at 7 days of inoculation/treatments under glasshouse conditions.

Furthermore, antioxidant enzyme activities have been shown to contribute significantly to plant resistance to abiotic and biotic stresses. Antioxidative enzymes have traditionally been linked to infections caused by plant pathogens. However, the response of antioxidant enzyme activities in wheat plants to Bipolaroxin-induced stress has been rarely/yet to be investigated. In the present study, the transcript levels of APx, POx, CAT, and SOD decreased several-fold in the susceptible line Agra Local challenged with the pathogen or infiltrated with Bipolaroxin 100 ng, followed by 75 ng and 50 ng in the first few hours, as compared to other treatments (data not shown). A slight increase in the levels of APx, POx, CAT, and SOD transcript was observed with the time increases (Figure 13a). However, the level of transcript (fold change) of APx, POx, CAT, and SOD was found to be lesser as compared to the resistant lines. Interestingly, significantly higher expression and transcript accumulation of APx, CAT, and SOD was recorded in the resistant cultivar Chirya3 as compared to Chinese Spring across the treatments (Figure 13b,c). However, transcript accumulation of POx was comparatively higher in Chinese Spring as compared to Chirya3 across the treatments. Among the different treatments, the highest transcript of these genes was reported in the plant leaves infiltrated with Bipolaroxin 100 ng, followed by 75 ng and 50 ng (Figure 13b,c).

#### 3.11.5. Effects on Symptom Development

**Figure 14:**
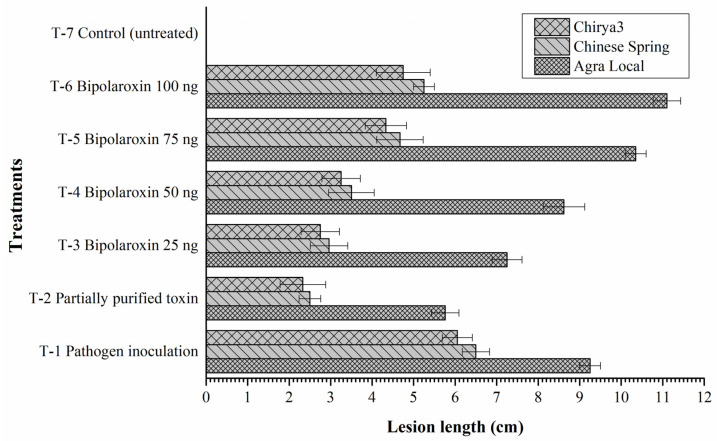
Effects of different treatments on lesion development in the susceptible and resistant lines/cultivars of wheat at 30 days of pathogen inoculation and infiltration of partially purified toxin obtained from *B. sorokiniana* as well as chemically synthesized Bipolaroxin. Data are mean (*n* = 5) and vertical lines represent the standard deviation (Mean ± SD).

The toxin Bipolaroxin produced by the pathogens in the plant during infection and its interaction with plant tissues are often linked with the symptom development. Figure 14 shows the difference in the lesion length formed on the leaves of susceptible line Agra Local versus resistant lines Chinese Spring and Chirya3. The results of the present investigation clearly demonstrate a positive correlation between the concentration of infiltrated Bipolaroxin and symptom development (lesion length). It was observed that a comparatively larger lesion was recorded on the susceptible check Agra Local as compared to the resistant lines Chinese Spring and Chirya3 across the treatments. In line with this observation, symptom development and lesion length (cm) were significantly decreased in the resistant lines Chinese Spring and Chirya3. Among the different treatments, significantly larger lesions were reported in the plant leaves infiltrated with Bipolaroxin 100 ng, followed by 75 ng and 50 ng in the susceptible line, Agra Local, and resistant lines, Chinese Spring and Chirya3 (Figure 14).

### 3.12. Histopathology

**Figure 15:**
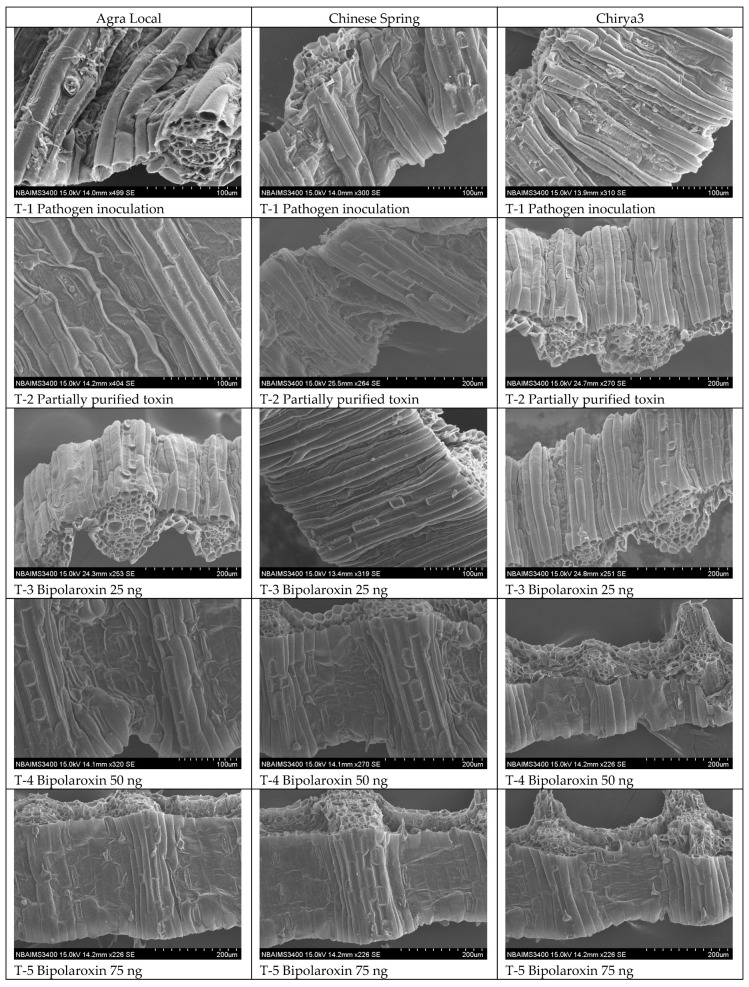

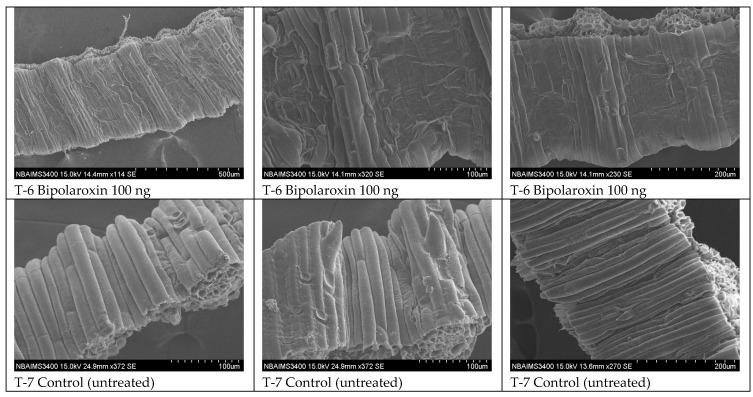
Effects of different treatments on cell wall integrity and tissue disintegration in the susceptible and resistant lines/cultivars of wheat at 30 days of under pathogen inoculation and infiltration of partially purified toxin obtained from *B. sorokiniana* as well as chemically synthesized Bipolaroxin.

Cell wall and cell membrane are the first layer of plant parts encountered by any biotic and abiotic stress and, therefore, play a crucial role in signaling, downstream processes, and maintaining cellular responses under these circumstances. Cell wall and tissue disintegration is often linked with symptom development and plant resistance. However, none of the studies demonstrated the effects of Bipolaroxin on tissue disintegration and downstream cellular responses in wheat. Like lesion development, pathogen infection and infiltration of crude toxin as well as different concentrations of Bipolaroxin (100, 75, 50, 25 ng) significantly affect the cell wall integrity and tissue integration. In the present investigation, scanning electron microphotographs (Figure 15) clearly show the differences in the level of cell wall shrinkage and tissue disintegration in the plant leaves infected by pathogen and infiltrated with crude toxin or Bipolaroxin 100 ng followed by 75 ng and 50 ng across the susceptible line Agra Local versus resistant lines Chinese Spring and Chirya3, as compared to untreated control plants (Figure 15).

#### Effects on Lignin Content

**Figure 16:**
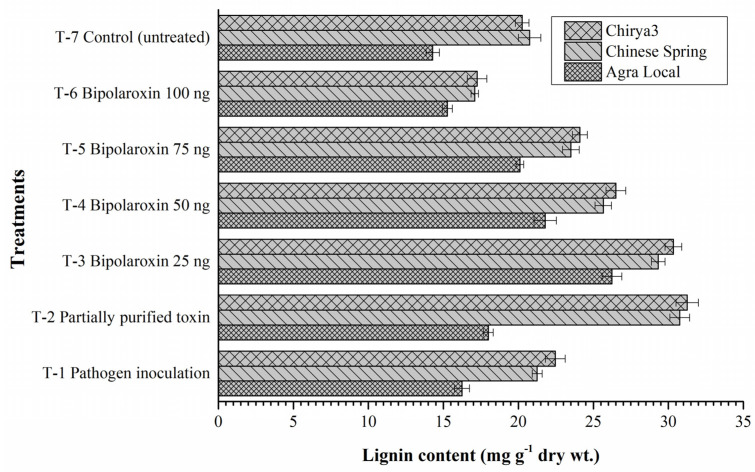
Effects of different treatments on lignin content in the susceptible and resistant lines/cultivars of wheat at 30 days under pathogen inoculation and infiltration of partially purified toxin obtained from *B. sorokiniana* as well as chemically synthesized Bipolaroxin. Data are mean (*n* = 5) and vertical lines represent the standard deviation (Mean ± SD).

In plants, phenolic polymer and cell wall component lignin is a key component and is shown to form a mechanical barrier against avirulent pathogens, thereby conferring disease resistance in plants. Lignification, in general, is induced during incompatible plant–pathogen interactions and lignin deposition is supposed to enhance disease resistance. In the present investigation, results revealed that lignin biosynthesis and deposition significantly varied in resistant and susceptible lines subjected to pathogen challenge and toxin infiltration. Pathogen challenge significantly reduced the lignin biosynthesis, and comparatively less lignin was reported in the plants treated with *B. sorokiniana* as compared to other treatments across the susceptible and resistant check. However, a comparatively increased amount of lignin was reported in the resistant cultivar Chirya3, followed by Chinese Spring and susceptible check, Agra Local, across the treatments (Figure 16). Interestingly, significant differences were recorded in the untreated controls of resistant lines Chirya3 and Chinese Spring, and the susceptible check Agra Local (Figure 16). Lignin content gradually decreased with an increase in the concentration of Bipolaroxin, which was also evident from histopathological studies (Figure 16).

## 4. Discussion

Mammalian heterotrimeric G proteins are key signal transducers composed of α, β, and γ subunits which are coupled with a plethora of receptors (G protein-coupled receptors; GPCRs) residing on the plasma membrane, and function as guanine nucleotide exchange factors (GEFs). They induce the Gα subunit to release the bound GDP and exchange it for GTP. The *Arabidopsis thaliana* gene *GPA1* encodes a Gα subunit (GPα1, 45 kDa), which is the most identical (36%) to Gαi1, a member of the mammalian Gi class of Gα subunits [16,17]. Moreover, the quest for the identification of a classical GPCR in plants is ongoing. It is also indicated that plant heterotrimeric G proteins are not activated by classical GPCRs. However, regarding the activated receptors, heterotrimeric G protein signaling in plants differs from that in animals. The heteromeric G proteins are widely conserved in plants [19]. In the plant system, heterotrimeric G protein has been implicated in auxin, gibberellin, and abscisic acid signaling, light responses, cell division, and ion-channel regulation [19,20]. They are also implicated in cell proliferation, defense, stomatal movement, sugar sensing, and response to phytohormones [45,46]. Different subunits of G-protein were involved in disease resistance against a variety of pathogens in different plants. The involvement of G-proteins in a plant-defined system was first described by Legendre et al. [47]. Recent literature shows that *Arabidopsis thaliana* is the only plant species where a considerable amount of progress has been made with respect to heterotrimeric G-protein. However, patho-physiological functions of Gα and Gβ in wheat are still largely undefined. The aim of the present investigation was to understand the molecular dialogues between Bipolaroxin and the Gα and Gβ subunits of the wheat heterotrimeric G-protein during host–pathogen interaction. In the present investigation, host–pathogen interaction mediated by cognate receptors and corresponding effector molecules can be explored through the molecular dynamics simulation (MD) studies [25,26,27]. Therefore, in this study, we employed a molecular modeling technique coupled with MD simulations to infer the probable mode of binding of Bipolaroxin to the Gα and Gβ subunits. Statistical approaches involving principal component analysis (PCA), ensemble clustering, and molecular mechanics-based binding free energy calculation were employed to elucidate the global motion and the energetic component that drives the interaction of the Gα and Gβ subunits to Bipolaroxin. The results presented in this study deliver the primary perceptions into the mode of binding and dynamics of Bipolaroxin with G-protein alpha subunits, which is expected to provide new avenues to host–pathogen interaction.

In order to explore the mode of binding of Bipolaroxin, we made an attempt to identify potential ligand-binding sites on the modeled Gα and experimental Gβ subunit structures of heterotrimeric G-protein through molecular docking followed by all-atoms MD simulation in an explicit solvent. Finally, for each complex, a representative structure was obtained from a cluster analysis of MD trajectories to infer the plausible mode of Bipolaroxin recognition. In the case of the Gα subunit, the cavity comprised of helical and Ras domain of the G-alpha subunit acts as the potential ligand-binding site, where nonbonded contacts (hydrogen bonds) mediated by Glu29, Ser30, Lys32, and Ala177 residues of the subunit act as crucial anchors for the ligand. However, in the case of the Gβ subunit, the residues, i.e., Lys256, Phe306, and Leu352, of the core beta strand domain participate in ligand interaction. Electrostatic surface potential map of the complex revealed that negatively charged surface residues dominate the ligand-binding pocket. As compared to docked conformation (Fig 3), the all-atom MD simulated cluster representation and displayed minor changes in the orientation of the ligand but stably formed crucial contacts to the ligand-binding cavity of both the subunits implicated in Bipolaroxin recognition (Figure 7). Taking all of the above into consideration, our study has unravelled the mode of ligand binding in the Gα and Gβ subunits of wheat heterotrimeric G-protein, which has advanced our comprehension of understanding the molecular basis of small-molecule recognition by host innate immune receptors. 

The in silico results prompt us to investigate the interaction of Bipolaroxin with susceptible and resistant lines of wheat. In planta investigations indicated that Bipolaroxin interacted with the cell membrane and affected the downstream signaling and biosynthesis processes, which is evident from the expression of key pathways associated with plant defense and histopathological studies. Demonstrable evidence showed that G-protein-mediated defense response includes the production of reactive oxygen species (ROS) including H_2_O_2_, hypersensitive response (HR), activation of NADPH oxidases, ion channels, and phospholipases [23,48,49]. In addition, antioxidant enzymes are capable of alleviating and/or neutralizing free radicals before they attack cellular components. It also showed that antioxidant enzyme activities in plants were stimulated to repair or to resist the damage caused by the collection of reactive oxygen species (ROS) due to biotic and abiotic stresses [50,51]. Results indicated that Bipolaroxin, even at lower concentrations, was implicated to be involved in the downregulation of key genes of the MKK and MAPK pathways, which consist of key genes involved in the signaling processes and sensing the elicitors/pathogen propagules at early stages, and thereby modulate plant defense accordingly. Additionally, a significantly lower expression (fold change) of these key genes was recorded in the susceptible line Agra Local, as compared to resistant lines Chirya3 and Chinese Spring. In addition, biochemical responses, including plant cellular antioxidant enzyme activities against disease stresses, are regarded as early warning indicators of injury, which are also related to resistance to sheath blight in rice [52]. 

Disease resistance in the wheat–*B. sorokiniana* pathosystem is conferred through the synthesis of a number of chemical compounds (lignin and phenolics) within the infected cells [24,50,51]. In response to *B. sorokiniana* infection, the host produces a range of secondary metabolites which are toxic to the pathogen and/or are inhibitory to the toxins produced by the pathogen [1,2]. The level of resistance to spot blotch in wheat has been correlated with a number of biochemical constituents. These include total content of phenolics, lignin, protein, reducing sugars, chlorophyll, and phenylalanine ammonia-lyase (PAL) [24,50,51,53,54,55,56]. The plant response to pathogen attack involves signaling mediated by the phytohormones such as salicylic acid (SA), jasmonic acid (JA), and ethylene. SA and JA are involved in defense signaling pathways of several pathosystems [57,58,59,60]. A number of key components of the defense signaling network in the wheat–*B. sorokiniana* pathosystem have already been identified over a period of time [24,50]. The interactions between host plants and pathogens involve bidirectional recognition. On the other hand, plants need to sense the foreign molecules delivered by pathogens to activate the plant’s innate immunity. Further, pathogens need to identify special target proteins to disrupt the plant immune system [61]. In plants, two layers of immunity have been proposed, pathogen-triggered immunity (PTI) and effector-triggered immunity [45]. While PTI functions in almost all the cases, it is the ETI that involves a specific response against disease and is, therefore, more important. ETI is more amplified and faster a system than PTI, which usually develops into the hypersensitive response (HR) leading the infected host cell to apoptosis. ETI involves the production of effectors by the pathogen that is perceived by the host through R genes. In the wheat–*B. sorokiniana* pathosystem, ETS has not been studied in detail, as the gene ToxA was discovered only recently in the genome of *B. sorokiniana* isolate from wheat [62].

## 5. Conclusions

The hemi-biotrophic fungus *B. sorokiniana* is a notorious pathogen of wheat which causes significant yield loss in wheat under warm and humid climatic conditions. This pathogen produces a number of toxins in the plants during infection processes in the host plants. These toxins have the ability to cause blightening of the plant tissues which ultimately led to reduction in photosynthetic area and plant mortality at early stages of crop growth. Among them, Bipolaroxin is one of key toxin produced by *B. Sorokiniana*. However, the exact mechanism by which this toxin interacts with host tissues is largely unexplored. In the present investigation, the authors have tried to elucidate the molecular dialogues between Bipolaroxin and the Gα and Gβ subunits of the wheat heterotrimeric G-protein during host–pathogen interaction. In order to explore the binding pattern of Bipolaroxin, an attempt was made to identify ligand’s potential binding sites on the modeled Gα and experimental Gβ subunit structures of heterotrimeric G-protein through molecular docking followed by all-atoms MD simulation in an explicit solvent. Further, *in planta* investigations clearly indicated that the application of Bipolaroxin led to downregulation of the signaling mechanisms and other cascades involved in the resistance development. A significant reduction in the accumulation of transcript of a key gene of MAPKs and phenylpropanoid pathways was encountered during qPCR analyses. Further, Bipolaroxin interacted with the Gα and Gβ subunits of G proteins, which corroborated the results of molecular docking and MD simulation. Histopathological studies and SEM microphotographs clearly showed the effects of Bipolaroxin on shrinking of cell wall and disintegration of host tissues. The present investigation gives a clue for further in-depth investigation which will certainly help in understanding the plant–pathogen integrations at a cellular and molecular level.

## Figures and Tables

**Table 1:** Model validation statistics of G-protein alpha subunit of *Triticum aestivum* using various structural evaluation servers.

Model Validation Servers	Model Quality Parameters	Validation Scores
Procheck (Ramachandran plot)	Most favored regions (%) Additional allowed regions (%) Generously allowed regions (%) Disallowed regions (%)	94.0 5.1 0.9 0.0
Verify 3D	Averaged 3D-1D score >= 0.2(%)	86.76
ERRAT	Overall quality (%)	82.22
ProSA	Z score	−8.05
ProQ	LG score Max Sub	5.347 0.509
Prove	Z score mean	−0.041
METAMQAP-II	GDT_TS	51.486

**Table 2:** Molecular docking results of Bipolaroxin with G-protein alpha and beta subunit of *Triticum aestivum* using AutoDock.

Target	Binding Energy (kcal/mol)	No. of H-bonds	H-bond Forming Residues	Average H-bond Distance (Å)	Hydrophobic Contacts
G-Alpha subunit	−8.19	6	Glu29, Ser30, Lys32, Ala177	2.75	Gly28, Gly31, Ser33, Thr34, Arg178, Val179, Thr181, Gly209,
G-Beta subunit	−7.47	7	Lys256, Phe306, Leu352	2.62	Trp74, Ala305, Phe306, Ile308, Leu350, Gly351 and Ser354

**Table 3:** The distribution of energy terms contributing to the binding free energy of each protein–ligand (G-alpha and -beta with systems Bipolaroxin). The free energy was computed using MM/PBSA method.

Protein–Ligand System	Van Der Waal Energy (kJ/mol)	Electrostatic Energy (kJ/mol)	Polar Solvation Energy (kJ/mol)	SASA Energy (kJ/mol)	Binding Energy (kJ/mol)
G-Alpha	−114.91 ± 0.85	−51.89 ± 1.43	143.51 ± 1.72	−12.25 ± 0.05	−35.54 ± 1.07
G-Beta	−134.61 ± 0.78	−73.12 ± 1.65	185.54 ± 2.53	−12.45 ± 0.06	−34.68 ± 1.39

## Data Availability

Not applicable.

## Supplementary Materials

The following supporting information can be downloaded at: https://www.mdpi.com/article/10.3390/antiox11091754/s1, Figure S1: The multiple sequence-structure alignment of G-alpha subunit of wheat with its close structural homologs from Arabidopsis, and Human aligned using ESPript software. The secondary structure displayed on the top was obtained from the crystal structure G alpha protein AtGPA1 from Arabidopsis thaliana (PDB ID: 2XTZ_A); Figure S2: The time dependent evolution of secondary structure elements of the G-α and G-β sub-unit complex systems during 50 ns MD simulation. The image was plotted using timeline viewer of VMD; Figure S3: The cross-correlation matrix of the C-α displacement of G-alpha (A) and beta (B) complex systems. Highly positive regions (red) designate a strong correlation in the movement of residues, whereas negative regions (blue) are associated with strong anti-correlated motion of the residues; Figure S4: Molecular representation of the top ranked clusters G alpha (A) and G-beta (C) subunit with Bipolaroxin rendered using LigPlot+, where the green dotted lines show the hydrogen bonds, residues with dark-red semi-circles forming hydrophobic contacts and residues labeled in green portrays the H-bond forming amino acids. Binding free energy per residue decomposition analysis of G-alpha (B) and G-beta sub-unit (D) complex used for binding free energy analysis. Per residue decomposition map displaying the contribution of each amino acids to the binding free energy of each complex system. The amino acids with a positive energy value impair the binding and vice versa Table S1: Inter-molecular contact analysis of the top ranked cluster of G-alpha and G-beta system with Biploaroxin; Table S2: qPCR primers of Gα and Gβ subunit of heteromeric G proteins; Table S3: qPCR primers of key genes of MAPKs pathways used in expression analyses; Table S4: qPCR primers of key genes of phenylpropanoid pathways used in expression analyses.
