# Supplementary material for: Novel Insights into Understanding the Molecular Dialogues between Bipolaroxin and the Gα and Gβ Subunits of the Wheat Heterotrimeric G-Protein during Host–Pathogen Interaction
Source: Antioxidants (Basel). 2022 Sep 5;11(9):1754. doi: 10.3390/antiox11091754 (PMC9495435; doi:10.3390/antiox11091754)
Supplement: Supplementary file 1 [file antioxidants-11-01754-s001.zip › antioxidants-1822448-supplementary.pdf]

## Supporting Information

## Novel insights into understanding the molecular dialogues between *Bipolaroxin* and Gα and Gβ subunit of the wheat heterotrimeric G-protein during host-pathogen interaction

Deepti Malviya[1†], Udai B. Singh[1†], Budheswar Dehury[2, †], Prakash Singh[3, †], Manoj Kumar[1], Shailendra Singh[1], Anurag Chaurasia[4], Manoj Kumar Yadav[5], Raja Shankar[6], Manish Roy[1], Jai P. Rai[7], Arup K. Mukherjee[8], I.S. Solanki[9], Arun Kumar[9], Sunil Kumar[1,10,*], Harsh V. Singh[1*]

**Table S1** Inter-molecular contact analysis of the top ranked cluster of G-alpha and G-beta system with Biploaroxin

| Protein-ligand system | Pairs | Distance | Type | Category of bonds |
|---|---|---|---|---|
| G-alpha-subunit with Biploaroxin | ARG75:HH21 - UNK371:O3 | 2.45614 | Hydrogen Bond | Conventional Hydrogen Bond |
| | ARG75:HH21 - UNK371:O3 | 2.45614 | Hydrogen Bond | Conventional Hydrogen Bond |
| | ARG178:HH11 - UNK371:O2 | 3.04711 | Hydrogen Bond | Conventional Hydrogen Bond |
| | ARG178:HH12 - UNK371:O2 | 2.7013 | Hydrogen Bond | Conventional Hydrogen Bond |
| | UNK371:H15 - LEU146:O | 2.73565 | Hydrogen Bond | Conventional Hydrogen Bond |
| | UNK371:O1 - LYS68:O | 2.9578 | Hydrogen Bond | Conventional Hydrogen Bond |
| | UNK371:O3 - CYS70:SG | 3.38649 | Hydrogen Bond | Conventional Hydrogen Bond |
| | UNK371:H15 - LEU146:O | 2.73565 | Hydrogen Bond | Conventional Hydrogen Bond |
| | UNK371:C18 - LYS68:O | 3.63798 | Hydrogen Bond | Carbon Hydrogen Bond |
| | UNK371:C18 - LYS68:O | 3.63798 | Hydrogen Bond | Carbon Hydrogen Bond |
| | LYS68 - UNK371 | 4.64792 | Hydrophobic | Alkyl |
| | UNK371:C11 - VAL148 | 5.11052 | Hydrophobic | Alkyl |
| | TYR116 - UNK371 | 4.92656 | Hydrophobic | Pi-Alkyl |
| Protein-ligand system | Pairs | Distance | Type | Category of bonds |
| G-beta-subunit with Biploaroxin | UNK381:O1 - ASP73:OD2 | 4.81706 | Electrostatic | Attractive Charge |
| | LYS256:HZ2 - UNK381:O4 | 2.06657 | Hydrogen Bond | Conventional Hydrogen Bond |
| | LYS256:HZ2 - UNK381:O4 | 2.06657 | Hydrogen Bond | Conventional Hydrogen Bond |
| | PHE306:H - UNK381:O3 | 2.37025 | Hydrogen Bond | Conventional Hydrogen Bond |
| | PHE306:H - UNK381:O3 | 2.37025 | Hydrogen Bond | Conventional Hydrogen Bond |
| | LEU352:H - UNK381:O3 | 2.75029 | Hydrogen Bond | Conventional Hydrogen Bond |
| | LEU352:H - UNK381:O1 | 2.56318 | Hydrogen Bond | Conventional Hydrogen Bond |
| | LEU352:H - UNK381:O3 | 2.75029 | Hydrogen Bond | Conventional Hydrogen Bond |
| | UNK381:H15 - LEU352:O | 2.2291 | Hydrogen Bond | Conventional Hydrogen Bond |
| | UNK381:O1 - LEU352:O | 2.6625 | Hydrogen Bond | Conventional Hydrogen Bond |
| | UNK381:H15 - LEU352:O | 2.2291 | Hydrogen Bond | Conventional Hydrogen Bond |
| | UNK381:O3 - PHE306 | 4.13086 | Hydrogen Bond | Pi-Donor Hydrogen Bond |
| | UNK381 - PRO76 | 4.61919 | Hydrophobic | Alkyl |
| | UNK381 - ILE308 | 4.60817 | Hydrophobic | Alkyl |
| | UNK381:C13 - LEU352 | 5.21716 | Hydrophobic | Alkyl |
| | PHE306 - UNK381 | 5.32001 | Hydrophobic | Pi-Alkyl |
| | PHE306 - UNK381:C13 | 4.1251 | Hydrophobic | Pi-Alkyl |

UNK: Biploaroxin

## Figures

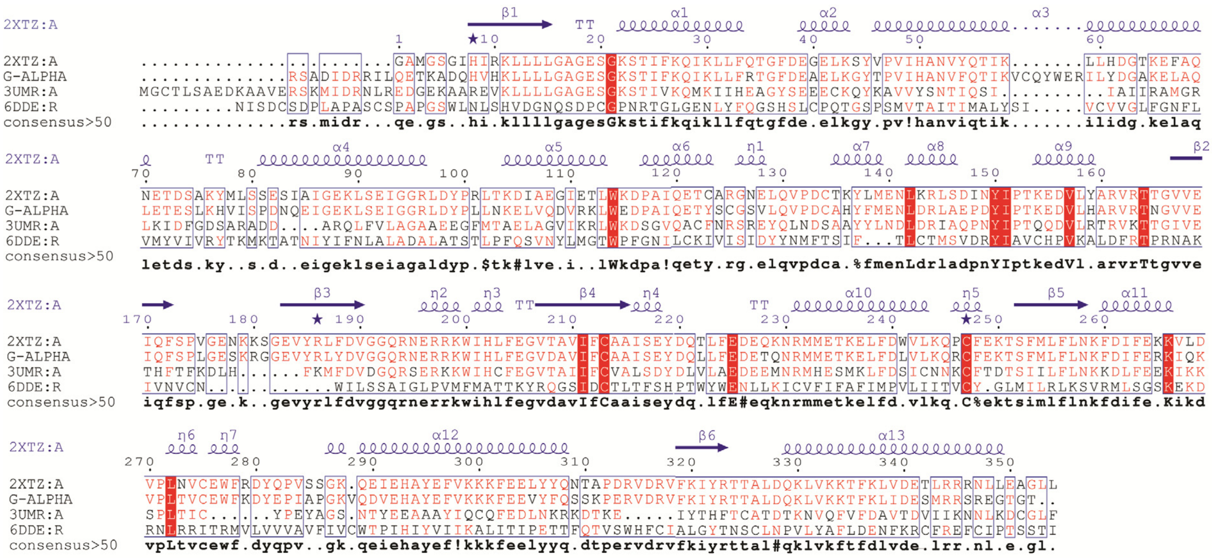

**Figures S1.** The multiple sequence-structure alignment of G-alpha subunit of wheat with its close structural homologs from Arabidopsis, and Human aligned using ESPript software. The secondary structure displayed on the top was obtained from the crystal structure G alpha protein AtGPA1 from Arabidopsis thaliana (PDB ID: 2XTZ_A).

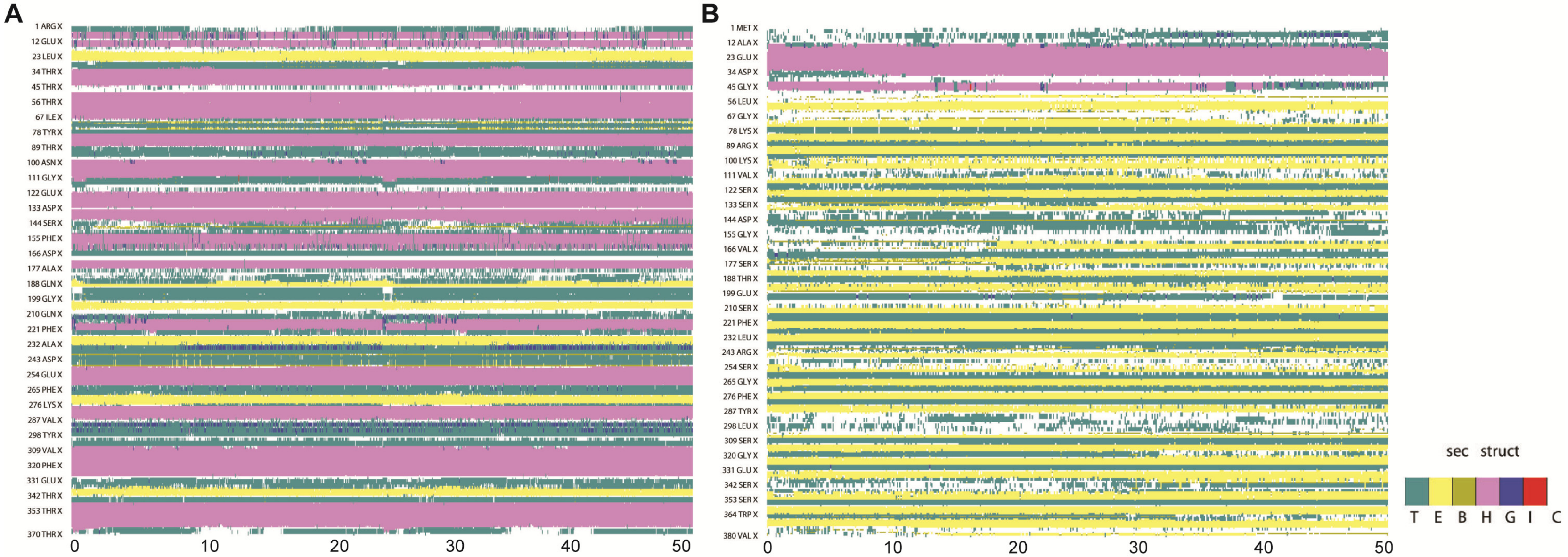


**Figure S2** The time dependent evolution of secondary structure elements of the G-α and G-β sub-unit complex systems during 50 ns MD simulation. The image was plotted using timeline viewer of VMD.

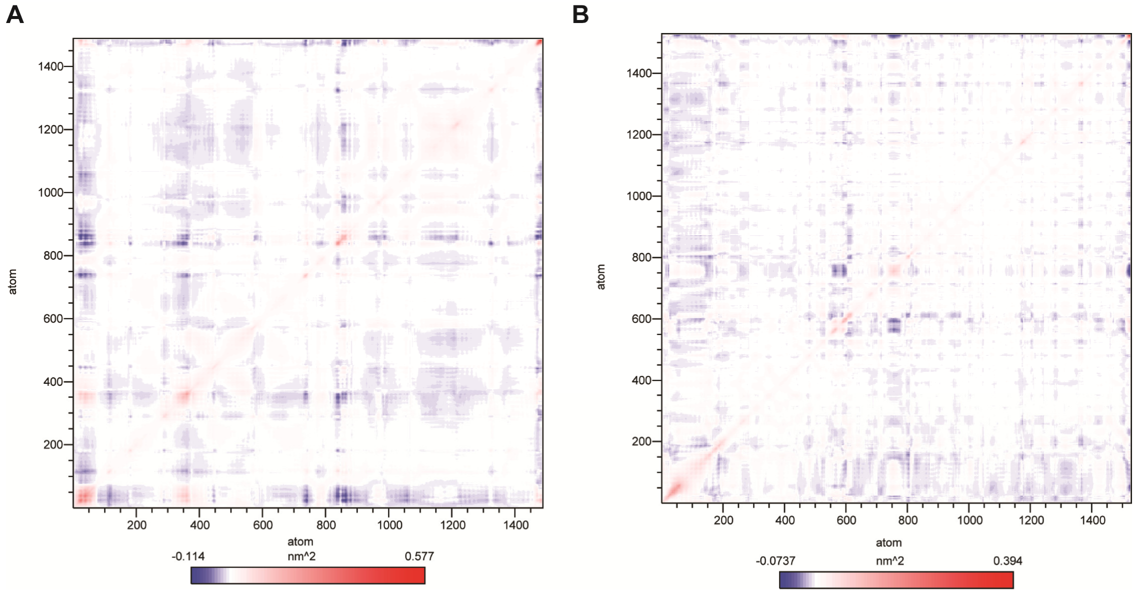


**Figure S3** The cross-correlation matrix of the C-α displacement of G-alpha (A) and beta (B) complex systems. Highly positive regions (red) designate a strong correlation in the movement of residues, whereas negative regions (blue) are associated with strong anti-correlated motion of the residues.

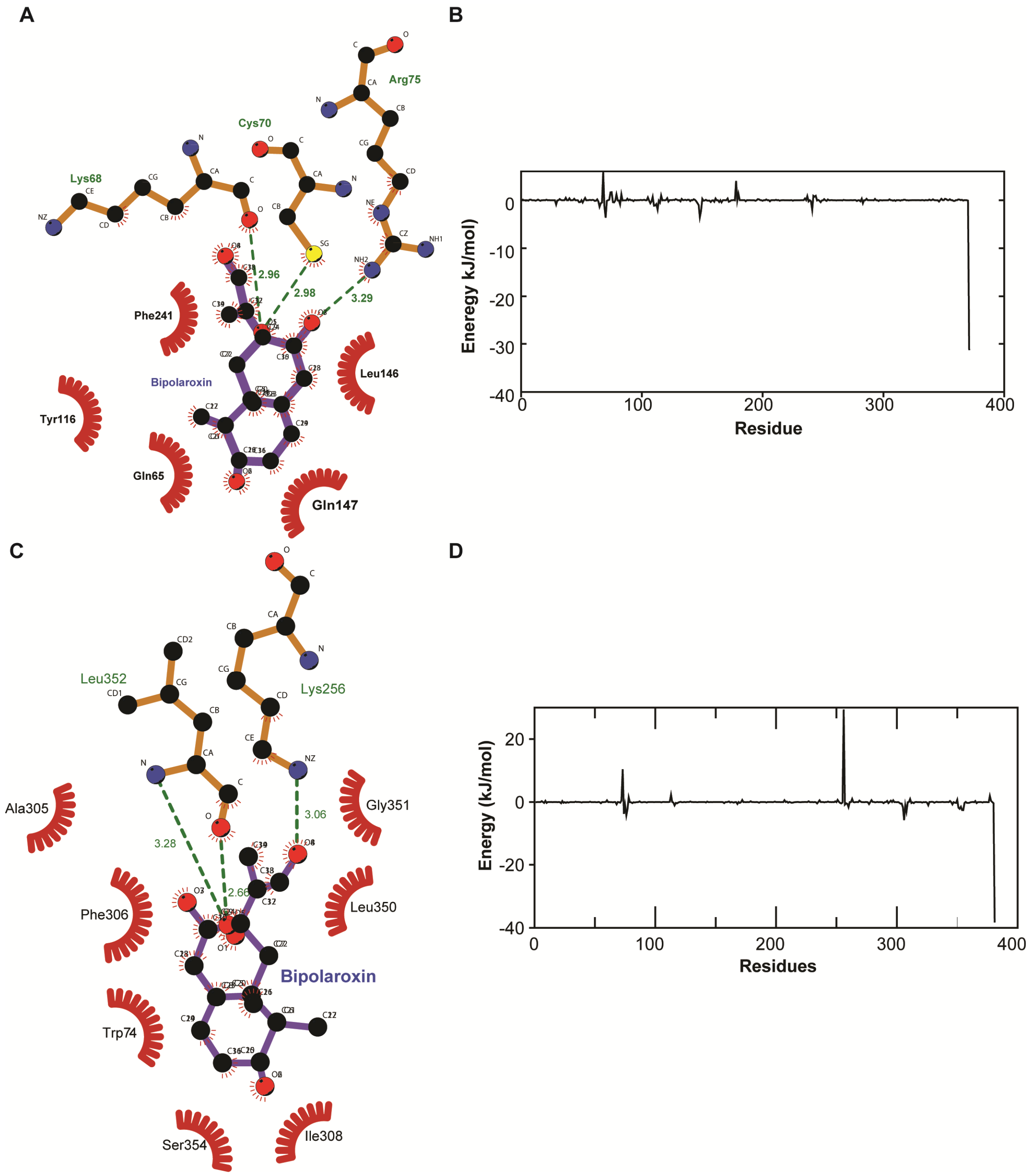


**Figure S4** Molecular representation of the top ranked clusters G alpha (**A**) and G-beta (**C**) subunit with Bipolaroxin rendered using LigPlot+, where the green dotted lines show the hydrogen bonds, residues with dark-red semi-circles forming hydrophobic contacts and residues labeled in green portrays the H-bond forming amino acids. Binding free energy per residue decomposition analysis of G-alpha (**B**) and G-beta sub-unit (**D**) complex used for binding free energy analysis. Per residue decomposition map displaying the contribution of each amino acids to the binding free energy of each complex system. The amino acids with a positive energy value impair the binding and vice versa.

S. Table 2. qPCR primers of Gα and Gβ subunit of heteromeric G proteins

| S. No. | Gene | Primer sequences |
|---|---|---|
| 1. | Triticum aestivum G protein beta subunit | F-TCCCTGATGGTCAGAGGTTC<br>R-AGCGTGTCCCACACATAACA |
| 2. | Triticum aestivum G protein beta subunit | F-TCCCTGATGGTCAGAGGTTC<br>R-GCGTGTCCCACACATAACAG |
| 3. | Triticum aestivum G protein beta subunit | F-AGACCAGGAAACTCGCTTGA<br>R-CGAACTGCCCGACTTGTAAT |
| 4. | Triticum aestivum G protein beta subunit | F-GACCAAACGTGTGTCCTGTG<br>R-CGAACTGCCCGACTTGTAAT |
| 5. | Triticum aestivum G protein beta subunit | F-TGACCAAACGTGTGTCCTGT<br>R-CGAACTGCCCGACTTGTAAT |
| 6. | Triticum aestivum G protein alpha subunit | F-ACTCGTGTGGAAGTGTGCTG<br>R-TCTCTCCAAGGGGGCTAAAT |
| 7. | Triticum aestivum G protein alpha subunit | F-AAGGTTCCTTTGACCGTGTG<br>R-TCCCTGGAGCGTCTCATACT |
| 8. | Triticum aestivum G protein alpha subunit | F-AAGGTTCCTTTGACCGTGTG<br>R-CCCTGGAGCGTCTCATACTC |
| 9. | Triticum aestivum G protein alpha subunit | F- AAGGTTCCTTTGACCGTGTG<br>R- CTCCCTGGAGCGTCTCATAC |
| 10. | Triticum aestivum G protein alpha subunit | F- AATGGGGTTGTGGAAATTCA<br>R- CGAACAGTTCCTTCGTCTCC |

S. Table 3. qPCR primers of key genes of MAPKs pathways used in expression analyses.

| S. No. | Gene | Primer sequences |
|---|---|---|
| 1. | *TaWRKY23* | F- GAGCGTAGACGTCAGCACCA |
| | | R- CACGGATGCTAATGGCCA |
| 2. | *TaWRKY29* | F- ATCCAACGGATCAAGAGCTG |
| | | R- GATGGGTTTCTGCCCGTATT |
| 3. | *TaWRKY70* | F- TCGACGACGCTGCTGCGACG |
| | | R- GCAGAGCTCCCTGGCGTGC |
| 4. | *TaMKK1* | F- GCGAGGCTCCTCCTATCAAG |
| | | R- CCATCTGCTTGCGTATGCTT |
| 5. | *TaMKK4* | F- GACATCAAGCCGTCCAACCT |
| | | R- CGTTGTAGTTGCCGTCGTTG |
| 6. | *TaMKK5* | F- CGCATCAACACCGACATCAA |
| | | R- GGAGTAGCAGATGGCGACC |
| 7. | *TaMKK6* | F- CAGCTTATCTCCGAGGAAAACG |
| | | R- TTGTGCCGCACTAGTTGGA |
| 8. | *TaMPK3* | F- GGAGATCAAGCTCCTCAGGC |
| | | R- ACTGGCAGTGTTCTTCCGAG |
| 9. | *TaMPK4* | F- TCGAGCCTGGGATTTCTTCG |
| | | R- GTCAACAGTGATGCGTCTGC |
| 10. | *TaMPK6* | F- CAGCTTATCTCCGAGGAAAACG |
| | | R- TTGTGCCGCACTAGTTGGA |
| 11. | *TaMPK20* | F- ATAAGCCTTCGCCTGACGAG |
| | | R- TTCGTAAGGCAGAACTGGACC |
| 12. | *TaMPK24* | F- CTCCGCCACCCAAACATAGT |
| | | R- CCTGCGGTGAGGTTCTCATT |
| 13. | *TaPR1* | F- GAACCAGGAAGTAATGGAGACGCCC |
| | | R- CAGGTGTTGGACCCGTAGTTGTAGTC |
| 14. | *TaPR-2* | F- AAGATGTTGCCTCCATGTTTGCAG |
| | | R- AAGTAGATGCGCATGCCGTTGAT |
| 15. | *TaPR-5* | F- GTTAATTAAGGTGCTCCTTCAATGGCG |
| | | R- TTGCGGCCGCTCATGGACAGAAGGTGATCTGGTA |
| 16. | *TaPR-5* | F- GTTAATTAA ATGGCGACCTCCGCGGTGCTC |
| | | R- TTGCGGCCGCCTACCGCCATTGAAGGAG |
| 17. | *TaPR-10* | F- AGTGCAAGTCCACCCTCATC |
| | | R- CTTGGCCTTGGTGATCTCAT |
| 18. | *TaTGA* | F- AAGCATTTCCAGTTGCTGCT |
| | | R- TCCAGGACAAGGAGATACCG |
| 19. | *TaNPR-1* | F- CCAAAACAGTCGAACTCGGCAA |
| | | R- GACGATGAGGAAGATGAAAGGGTTG |
| 20. | *TaAPx* | F- GGCTAATGCTGGTCTTGTGA |
| | | R- GCACTGGCAAGTTGAAACAG |
| 21. | *TaCAT* | F- GAGTCGCTCCACATGTTCAC |
| | | R- TTCACCAGCGTGTAGGTGTT |
| 22. | *TaSOD* | F- GCCCATTTTGGCGAGTGTTT |
| | | R- GACAAGAGACAAGCCCGGAA |
| 23. | *TaPOx* | F- GGCATGGAACAAAACGCTAT |
| | | R- TGATACTCTTACGGCGACGA |
| 24. | *Beta-tubulin* | F- GCTCACATCTCGTGGGTCACAGA |
| | | R- CGCCAGTGTACCAATGCAAGAAA |
| 25. | *β-actin* | F- TGCTATCCTTCGTTTGGACCTT |
| | | R- AGCGGTTGTTGTGAGGGAGT |
| 26. | *TEF1* | F- GGTGATGCTGGCATAGTGAA |
| | | R- GATGACACCAACAGCCACAG |

S. Table 4. qPCR primers of key genes of phenylpropanoid pathways used in expression analyses.

| S. No. | Gene | Primer sequences |
|---|---|---|
| 1. | *Triticum aestivum* phenylalanine ammonia-lyase [EC:4.3.1.24] | F- TCATGTTTGCCCAATTCTCA<br>R- CTGGACATGGTTGGTCACAG |
| 2. | *Triticum aestivum* phenylalanine ammonia-lyase [EC:4.3.1.24] | F- GCTCATGTTTGCCCAATTCT<br>R- CTGGACATGGTTGGTCACAG |
| 3. | *Triticum aestivum* phenylalanine ammonia-lyase [EC:4.3.1.24] | F- CTGTGACCAACCATGTCCAG<br>R- TGTCTTCCTAGCCACCGTCT |
| 4. | *Triticum aestivum* phenylalanine/tyrosine ammonia-lyase [EC:4.3.1.25] | F- CTGTTCTCTCCGAGGTCCTG<br>R- TTGAGCTGCGTGTCAATCTC |
| 5. | *Triticum aestivum* phenylalanine/tyrosine ammonia-lyase 7 (PAL7) [EC:4.3.1.25] | F- CCACTGTTGACGGGAAGAAT<br>R- CAGGACCTCGGAGAGAACAG |
| 6. | *Triticum aestivum* phenylalanine/tyrosine ammonia-lyase 6 (PAL6) [EC:4.3.1.25] | F- CCACTGTTGACGGGAAGAAT<br>R- CAGGACCTCGGAGAGAACAG |
| 7. | *Triticum aestivum* cinnamoyl-CoA reductase [EC:1.2.1.44] | F- GTTCACCCCTGTGCAAGAAT<br>R- TGCGGAAGCCTATCAAAGTT |
| 8. | *Triticum aestivum* cinnamoyl-CoA reductase [EC:1.2.1.44] | F- ACCTTGGCATGAAGTTCACC<br>R- TGCGGAAGCCTATCAAAGTT |
| 9. | *Triticum aestivum* cinnamyl-alcohol dehydrogenase [EC:1.1.1.195] | F- ACACCGACCTCCACTTCATC<br>R- CCCAGAAGATGCCGTTGTAT |
| 10. | *Triticum aestivum* cinnamyl-alcohol dehydrogenase [EC:1.1.1.195] | F- GTCTCTACCGACGGGATCAA<br>R- TGGGCTGAAAATGAAGAAGG |
| 11. | *Triticum aestivum* 4-coumarate--CoA ligase [EC:6.2.1.12] | F- TACTCCTGTCCCATCCTTCG<br>R- AGCATCCGGAGCTTCTTGTA |
| 12. | *Triticum aestivum* 4-coumarate--CoA ligase [EC:6.2.1.12] | F- CTGGAGGCCATACTCCTGTC<br>R- AGCATCCGGAGCTTCTTGTA |
| 13. | *Triticum aestivum* ferulate-5-hydroxylase [EC:1.14.-.-] | F- TCGACGAGTTCATCGACAAG<br>R- GCCAAACATCACATCCATGA |
| 14. | *Triticum aestivum* ferulate-5-hydroxylase [EC:1.14.-.-] | F- TCGCCTACCTCACCTACGAC<br>R- CGGAAGATGACGTTCTTGGT |
| 15. | *Triticum aestivum* class II peroxidase-like [EC:1.11.1.7] | F- TAGCACGCTGCACAAACTTC<br>R- CCATTGAAGAGCTCCTGGTC |
| 16. | *Triticum aestivum* class II peroxidase-like [EC:1.11.1.7] | F- ATTGCTGTCCTCTCGCTCAT<br>R- AGCAGTCGTGGAAGAAGAGG |
| 17. | *Triticum aestivum* peroxidase [EC:1.11.1.7] | F- CTCTCCGGATCTCACACCAT<br>R- CTTGGCAACCAGGTTCTTGT |
| 18. | *Triticum aestivum* peroxidase [EC:1.11.1.7] | F- ACACGCGCGCTCTATAGTCT<br>R- GGCACGAGCTCGAGTAGAAC |
